# Establishment of xenogeneic serum-free culture methods for handling human dental pulp stem cells using clinically oriented in-vitro and in-vivo conditions

**DOI:** 10.1186/s13287-017-0761-5

**Published:** 2018-02-03

**Authors:** Mai Mochizuki, Taka Nakahara

**Affiliations:** Department of Developmental and Regenerative Dentistry, School of Life Dentistry at Tokyo, The Nippon Dental University, 1-9-20 Fujimi, Chiyoda-ku, Tokyo, 102-8159 Japan

**Keywords:** Human dental pulp stem cells, Xenogeneic serum-free culture, Cell isolation, Stem cell characterization, Chromosomal stability, Cytotoxic susceptibility, In-vivo transplantation, Cryopreservation, Cell cycle, Apoptosis

## Abstract

**Background:**

Currently, ex-vivo handling of stem cells, including transport after harvest and therapeutic preparation, is generally done in culture media containing fetal bovine serum (FBS), which promotes cell attachment, proliferation, and differentiation. However, because of safety concerns associated with the use of FBS, including potential transmission of zoonotic agents and transplant rejection because of the incorporation of foreign proteins into the stem cells, there is a need for xenogeneic serum-free culture media for clinical handling of stem cells.

**Methods:**

Dental pulp stem cells were derived from wisdom teeth donated by eight healthy volunteers and cultured in xenogeneic serum-free culture medium (XFM) or xenogeneic serum-containing culture medium (SCM). Cells were subjected to morphological, proliferation, karyotype, differentiation, marker expression, cryopreservation, and cytotoxic susceptibility analyses in vitro, as well as transplantation in vivo.

**Results:**

In primary culture, XFM cells showed lower adhesion and slightly different morphology, although the single-cell size was similar to that of SCM cells. XFM cells exhibited typical mesenchymal stem cell (MSC) characteristics in vitro and in vivo, including marker gene/protein expression, trilineage differentiation potential, and hard, osteo-dentin tissue formation. Additionally, XFM cells maintained a normal karyotype in vitro and nontumorigenic potential in vivo; however, XFM cells were more susceptible to H_2_O_2_ and ultraviolet cytotoxic stimuli. XFM cells formed a multilayered structure showing excessive cell death/division in contrast to the monolayered structure of SCM cells when reaching overconfluence. Proliferation was disrupted in overconfluent XFM cells, and these cells could not be subcultured. Dimethyl sulfoxide-free cryopreserved XFM cells yielded similar results in all of the experiments.

**Conclusions:**

This study is the first reporting successful isolation and expansion of an MSC population from donor-derived tissue (dental pulp) under xenogeneic serum-free culture conditions, as well as the application of cryopreservation, using a research strategy based on clinically oriented in-vitro and in-vivo experiments.

**Electronic supplementary material:**

The online version of this article (doi:10.1186/s13287-017-0761-5) contains supplementary material, which is available to authorized users.

## Background

Various somatic stem cell-based therapies involving cell isolation, expansion, and application for treatment have been developed, with part of these subsequently introduced into clinical practice [1, 2]. The development and application of a novel cell therapy requires ex-vivo manipulation and therapeutic preparation of autologous stem cells [3–5]. Therefore, ensuring steady supplies of stem cells of various types and both quality and safety controls for the cells are essential for their wide application in stem cell-based tissue engineering and regenerative medicine [6, 7].

Mesenchymal stem cells (MSCs) isolated from dental pulp tissue of extracted teeth, which are generally discarded after dental treatments, represent autologous stem cell candidates for future cell therapies. Dental pulp stem cells (DPSCs) possess extensive proliferation ability, which is superior to that of iliac bone-marrow-derived MSCs [8, 9], and multipotency into a variety of cell lineages, such as osteoblasts, odontoblasts, adipocytes, chondrocytes, neurons, endothelial cells, myocytes, and hepatocytes in vitro [8–15]. Moreover, in-vivo studies reported the effective application of DPSCs in several animal models of systemic diseases [16–19]. More recently, clinical studies reported the therapeutic use of DPSCs for bone augmentation in tooth-extraction defects [20] and for dental pulp regeneration in caries-affected, pulpectomized teeth [21]. Therefore, DPSCs are currently regarded as a valuable, promising MSC type among somatic stem cells with clinical potential.

Fetal bovine serum (FBS) is generally added to culture media used for MSC isolation and expansion in vitro. The identified and unidentified contents of FBS facilitate various cell behaviors, such as cell attachment, proliferation, and differentiation, during cell culture ex vivo. However, there are several safety concerns for clinical application of MSCs expanded ex vivo with FBS, including potential risks of immune/allergic reactions and transmission of harmful agents, such as prions, viruses, or zoonotic microorganisms, to the host. Moreover, proteins and peptides within FBS are believed to be incorporated into the cultured cells and subsequently transferred into the host through the cell-transplantation process, resulting in the rejection of transplanted cells [22, 23]. Hence, recent studies reported the development and application of xenogeneic serum-free culture media using several MSC types for the purpose of clinical use [24–26].

Although several FBS-free culture media are commercially available, it is widely accepted that the use of FBS is essential in transporting harvested tissue samples to the laboratory and when isolating MSCs in primary culture. However, culturing primary cells in conventional FBS-containing medium even once raises the aforementioned safety concerns. In the current study, we thoroughly investigated the isolation and expansion of DPSCs under FBS-free culture conditions, as well as the application of cryopreservation, using a research strategy based on clinically oriented in-vitro and in-vivo experiments. The aim of this study was to establish practical and reliable methods for handling DPSCs under xenogeneic serum-free culture conditions to enhance clinical application of donor-derived MSCs.

## Methods

### Isolation and expansion of DPSCs using FBS-free culture medium

Wisdom teeth were donated by eight healthy volunteers aged 20–37 years. The teeth were transported at 4 °C in FBS-free basic culture medium comprising Dulbecco’s modified Eagle’s medium/Ham’s nutrient mixture F12 (DMEM/F12), 100 μM glutamate, 0.1% MEM nonessential amino acids, 50 U/ml penicillin, 50 μg/ml streptomycin, and 0.25 mg/ml Fungizone (all from Thermo Fisher Scientific, Waltham, MA, USA). The collection of dental pulp tissue from extracted teeth and DPSC isolation were performed as described previously [8]. Briefly, the dental pulp tissue harvested from the teeth was minced and digested in a solution containing 3 mg/ml collagenase type I (Merck KGaA, Darmstadt, Germany) and 4 mg/ml dispase (Wako Pure Chemical Industries, Osaka, Japan) for 1 h at 37 °C. After passing the cell suspension through a 70-μm cell strainer, the single-cell suspension was divided equally for cultivation in two types of culture media as follows: xenogeneic serum-free culture medium (XFM) (PRIME-XV® MSC Expansion XSFM; Irvine Scientific, Santa Ana, CA, USA); and the aforementioned basic culture medium supplemented with 15% FBS, designated xenogeneic serum-containing culture medium (SCM). The culture dishes/plates for XFM cultivation were precoated with PRIME-XV® human fibronectin (Irvine Scientific) in primary and subsequent cell cultures according to the manufacturer’s recommendations. Cell cultures were maintained at 37 °C in a humidified atmosphere containing 4.7% carbon dioxide in air, and both culture media were changed every 3 or 4 days. At 80% confluence, the cultured cells were detached using 0.25% trypsin (Becton Dickinson, Franklin Lakes, NJ, USA)–0.02% ethylenediaminetetraacetic acid (EDTA; Dojindo, Kumamoto, Japan) and were subcultured at 5 × 10^3^ cells/cm^2^ in 60-mm culture dishes (Thermo Fisher Scientific). Cells at passage 3 or 4 were used for experiments, except for karyotype analysis (passage 10). All experiments were repeated independently at least three times.

### Dimethyl sulfoxide-free cryopreservation of DPSCs

Part of the cells subcultured in XFM (“XFM cells”) at passage 1 were stored in dimethyl sulfoxide (DMSO)-free cryopreservation medium (CryoScarless® DMSO-free; BioVerde, Kyoto, Japan), at −80 °C. After 1–3 months, the cryopreserved XFM cells were recovered and cultured until passage 3, followed by characterization.

### Cell morphological and morphometric analyses

To evaluate subconfluent cells under the culture (adherent) condition, XFM and SCM cells were seeded at 5 × 10^3^ cells/cm^2^ into 60-mm culture dishes and cultured until subconfluence. Cells were fixed with 4% paraformaldehyde (PFA) and permeabilized with 1% Triton X-100 for 10 min. After washing with calcium and magnesium-free phosphate-buffered saline (PBS), the cells were stained F-actin with Phalloidin-iFluor 488 conjugate (AAT Bioquest, Sunnyvale, CA, USA) and mounted with Vectashield hard-set mounting medium containing 4′,6-diamidino-2-phenylindole (DAPI; Vector Laboratories, Burlingame, CA, USA). Five hundred cells were selected randomly under a Biorevo BZ-9000 microscope (Keyence, Osaka, Japan), and the adhesive area of each cell was measured using image analysis software (BZ-H2A; Keyence).

To evaluate cells under floating (nonadherent) conditions, XFM and SCM cells at subconfluence were detached by trypsin–EDTA treatment and fixed with 4% PFA. After washing with PBS, the cells were analyzed by measuring the transmitted light using PERFLOW® Sort V-4cTS2L-D (Furukawa, Chiba, Japan). The data were analyzed with FlowJo software (version 10; TreeStar, Ashland, OR, USA).

To observe fine cell morphology, both types of cells were seeded at 5 × 10^3^ cells/cm^2^ into 35-mm glass-bottom culture dishes (Greiner Bio-One, Frickenhausen, Germany). The cells were fixed with 4% PFA at confluence or overconfluence and stained with Phalloidin-iFluor 488 conjugate and DAPI as already described. Fluorescence and phase-contrast images were obtained using an inverted fluorescence microscope (IX71; Olympus, Tokyo, Japan). For three-dimensional (3D) analysis of confluent cell cultures, Z-stack images were obtained with a confocal laser-scanning microscope (LSM 700; Carl Zeiss, Oberkochen, Germany), and 3D images were reconstituted from the Z-stack data with image analysis software (IMARIS version 7.11; Bitplane, Zurich, Switzerland).

### Cell proliferation analyses

XFM and SCM cells (5 × 10^3^ cells/cm^2^) were seeded into 12-multiwell plates (SumitomoBakelite, Tokyo, Japan) and cultured for 14 days. The cultured cells were detached using trypsin–EDTA and routinely counted in triplicate with a hemocytometer every 2 days. The population doubling time (PDT) was calculated using the following formula:

$$ \mathrm{PDT}=\frac{\left(t-{t}_0\right) lo{g}_2\;}{\mathit{\log}\left(N-{N}_0\right)} $$

where *t* is time (hours), *N* is the number of cells, and *N*_0_ and *N* represent the number of cells at *t*_0_ and *t*, respectively.

### Electrochemiluminescence immunoassay

The levels of carcinoembryonic antigen (CEA), squamous cell carcinoma antigen (SCC), and neuron-specific enolase (NSE), which are common neoplastic/tumorigenic transformation markers, within conditioned media collected from XFM and SCM cultures on day 14 were measured by automated electrochemiluminescence immunoassay (ECLIA) in a commercial laboratory (SRL, Tokyo, Japan).

### Karyotype analysis

Chromosomal G-band analysis of XFM and SCM cells at passage 10 was performed at the Nihon Gene Research Laboratories (Sendai, Japan). Briefly, subconfluent cells were subjected to karyotype analysis, and Giemsa-stained cell metaphase spreads were prepared according to standard cytogenetic methods and selected randomly for analysis. Chromosome numbers were counted in 50 metaphase spreads, and G-banding patterns were assessed in 20 metaphase spreads.

### Cell-surface-marker expression analysis by flow cytometry

Subconfluent cells were collected with trypsin–EDTA and fixed with 4% PFA for 20 min at room temperature. After washing with PBS, the fixed cells were labeled with fluorescein isothiocyanate-conjugated antibodies against human CD14 (clone M5E2), CD90 (clone 5E10), and CD105 (clone 266) (Becton Dickinson), CD34 (clone 581) and CD44 (clone J.173) (Beckman Coulter, Fullerton, CA, USA), STRO-1 (clone STRO-1; Santa Cruz Biotechnology, Dallas, TX, USA), and phycoerythrin-conjugated mouse immunoglobulin (Ig) G1 (clone MOPC-21; Becton Dickinson). Flow-cytometric analysis was performed using a Guava™ EasyCyte HT system and Guava™ Express Plus software (version 2.7; Merck KGaA).

### Reverse transcription polymerase chain reaction

To detect the gene-expression profiles of MSC, osteogenic, and adipogenic markers, total RNA was extracted from XFM and SCM cultures at passage 3. To explore the time-course expression of apoptosis-related and cell cycle-related genes, XFM and SCM cells (5 × 10^3^ cells/cm^2^) were seeded into 12-multiwell plates and cultured for 14 days. Total RNA was extracted from the cultures every 2 days from days 6 to 14. RT-PCR was performed according to our previous report [27]. The semiquantitative densitometric analysis of the observed signals was performed using ImageJ software (version 1.48; National Institutes of Health, Bethesda, MD, USA) and expressed as a ratio to β-actin signal intensity. Primer sequences and PCR conditions are presented in Table 1.

**Table 1:** Primer sequences and amplification conditions for RT-PCR analysis

Gene	Primer sequences, 5′–3′		Product size (bp)	Annealing temp. (°C)	PCR cycles	GenBank accession number
*Vimentin*	S:	GGGACCTCTACGAGGAGGAG	200	55	35	NM_003380
	A:	CGCATTGTCAACATCCTGTC				
*Runx2*	S:	CCCCACGACAACCGCACCAT	292	55	35	NM_004348
	A:	GTCCACTCCGGCCCACAAATC				
*Collagen I*	S:	CCAAATCTGTCTCCCCAGAA	214	55	35	NM_000088
	A:	TCAAAAACGAAGGGGAGATG				
*Nestin*	S:	AACAGCGACGGAGGTCTCTA	220	55	35	NM_006617
	A:	TTCTCTTGTCCCGCAGACTT				
*Nanog*	S:	ACCTTCCAATGTGGAGCAAC	199	55	35	NM_024865
	A:	GAATTTGGCTGGAACTGCAT				
*Oct3/4*	S:	GACAGGGGGAGGGGAGGAGCTAGG	144	60	35	NM_001173531
	A:	CTTCCCTCCAACCAGTTGCCCCAAAC				
*Sox2*	S:	AACCCCAAGATGCACAACTC	152	60	40	NM_003106
	A:	CGGGGCCGGTATTTATAATC				
*OCN*	S:	GCTGAGTCCTGAGCAGCAG	323	60	30	NM_199173
	A:	CGATAGGCCTCCTGAAAGC				
*DSPP*	S:	CATTTGGGCAGTAGCATGGG	170	55	35	NM_014208
	A:	CACCTTCATGCACCAGGACA				
*PPARγ*	S:	TCGGATCCCTCCTCGGAAAT	399	60	35	AB565476
	A:	GCAGGCTCCACTTTGATTGC				
*FABP4*	S:	TGGGCCAGGAATTTGACGAA	212	60	35	NM_001442
	A:	ACGTCCCTTGGCTTATGCTC				
*Bcl-2*	S:	GGTGAACTGGGGGAGGATTG	214	60	35	NM_000633
	A:	GAAATCAAACAGAGGCCGCA				
*Bax*	S:	AAGAAGCTGAGCGAGTGTCTC	338	60	35	NM_001291428
	A:	AGTCGCTTCAGTGACTCGG				
*p53*	S:	TACCAGGGCAGCTACGGTTT	572	55	25	AB082923
	A:	CCTTTCTTGCGGAGATTCTCT				
*p21*	S:	TCAGAACCGGCTGGGGATGT	554	60	35	NM_001291549
	A:	AGATGTAGAGCGGGCCTTTG				
*p16*	S:	CCCAACGCACCGAATAGT	135	65	35	XM_011517676
	A:	CACGGGTCGGGTGAGAGT				
*β-actin*	S:	GGACTTCGAGCAAGAGATGG	234	60	30	NM_001101
	A:	AGCACTGTGTTGGCGTACAG				

*RT-PCR* reverse transcription polymerase chain reaction, *S* sense, *A* antisense, *Runx2* runt-related transcription factor 2, *Oct3/4* POU class 5 homeobox 1 (POU5F1), *SOX2* sex determining region Y-box 2, *OCN* osteocalcin, *DSPP* dentin sialophosphoprotein, *PPARγ* peroxisome proliferator-activated receptor gamma, *FABP4* fatty acid binding protein 4, *Bcl-2* B-cell lymphoma 2, *BAX* B-cell lymphoma-2 associated X

### In-vitro multilineage differentiation

In-vitro multilineage differentiation experiments (osteogenic, adipogenic, and chondrogenic) were performed according to our previous report [27]. Briefly, for osteogenic and adipogenic differentiation, XFM and SCM cells were seeded at 1 × 10^5^ cells/well in six-well plates (SumitomoBakelite). Both types of cells were cultured in osteogenic induction medium—α-modified minimal essential medium (α-MEM; Wako Pure Chemical) containing 10% FBS, 10 nM dexamethasone (Merck KGaA), 10 mM β-glycerophosphate (Merck KGaA), and 100 μM l-ascorbate-2-phosphate (Wako Pure Chemical)—or adipogenic induction medium—α-MEM containing 10% FBS, 0.5 mM 3-isobutyl-1-methylxanthine (Merck KGaA), 0.5 μM hydrocortisone (Wako Pure Chemical), and 60 μM indomethacin (Merck KGaA). As a control, the cells were cultured in α-MEM supplemented with 10% FBS lacking the osteogenic or adipogenic supplements. After 4 weeks of differentiation, the mineralized deposits were visualized by Alizarin Red S staining, and intracellular accumulation of lipid droplets was visualized by Oil Red O staining. For chondrogenic differentiation, approximately 1 × 10^6^ cells were centrifuged at 430 × *g* for 5 min to form a pellet. The pellets were cultured in chondrogenic induction medium: DMEM/F12 containing 10% FBS, 10 ng/ml of transforming growth factor-β1 (Peprotech, Oak Park, CA, USA), 1% ITS + 1 supplement (Merck KGaA), and 50 mM l-ascorbate-2-phosphate (Wako Pure Chemical). After 4 weeks of differentiation, the pellets were fixed in 4% PFA, embedded in paraffin, and sectioned at a thickness of 5 μm for histological analysis. Chondrogenic differentiation was determined by staining with Alcian Blue (Merck KGaA) and Safranin-O (Waldeck GmbH & Co. KG, Münster, Germany) and by immunohistochemistry. The rabbit polyclonal anti-type II collagen primary antibody (1:50; Santa Cruz Biotechnology) was used. The sections were examined using a Biorevo BZ-9000 microscope (Keyence).

### In-vitro assessment of cell damage induced by extrinsic cytotoxic stimuli

To evaluate cell susceptibility to extrinsic stimuli, XFM and SCM cells (5 × 10^3^ cells/cm^2^) were seeded into 12-multiwell plates and treated with 1 μM staurosporine (Wako Pure Chemical Industries) or 100 μM hydrogen peroxide (H_2_O_2_; Wako Pure Chemical Industries) for 6 h at 37 °C, or were exposed to ultraviolet (UV) radiation using a UV transilluminator (UVP, Upland, CA, USA) for 30 min at room temperature. Staurosporine, which is a known chemical inducer of apoptosis, was used as a positive control for cell-damage induction. Cell damage was evaluated by flow cytometry using an Annexin V/propidium iodide (PI) system described previously [28], with minor modifications. Briefly, the treated cells were stained with 100 μl of Guava Nexin® reagent (Merck KGaA) for 20 min at room temperature in the dark and subjected to flow cytometry in the Guava™ EasyCyte HT system equipped with Guava™ InCyte software (Merck KGaA). The data were analyzed with FlowJo software (TreeStar). Annexin V-positivity and PI-negativity indicated the fraction of early apoptotic cells and Annexin V/PI-double positivity indicated the fraction of late apoptotic cells. We considered that both fractions represented damaged cells [28]. The results were expressed as the ratio of the number of damaged cells to the number of nondamaged cells.

### In-vivo transplantation and histological evaluation

DPSCs expanded using XFM or SCM were transplanted subcutaneously under the dorsal skin of 6-week-old female nude mice (BALB/C-nu/nu; Nihon Clea, Tokyo, Japan). The preparation of cell-loaded hydroxyapatite/β-tricalciumphosphate (HA) constructs and the transplantation procedure were performed according to our previous report [28]. HA constructs alone were included as a control. All constructs were collected 16 weeks after transplantation. The samples were fixed with 4% PFA, decalcified, embedded in paraffin, and sectioned serially at 5-μm thickness [29]. The serial sections were subjected to hematoxylin and eosin (HE), Masson’s trichrome (MT), and immunohistochemical staining. Immunohistochemistry was conducted as described previously [27, 28]. Mouse monoclonal anti-human-specific vimentin (1:10,000; Merck KGaA) and rabbit polyclonal anti-dentin sialoprotein (anti-DSP) (1:200; Santa Cruz Biotechnology) were used as primary antibodies. All slides were examined using a Biorevo BZ-9000 microscope (Keyence).

### Terminal deoxynucleotidyl transferase-mediated deoxyuridine triphosphate nick-end labeling staining

To examine apoptotic cells within overconfluent monolayer/multilayer cultures and cell aggregates derived from subcultured overconfluent XFM cells, terminal deoxynucleotidyl transferase-mediated deoxyuridine triphosphate nick-end labeling (TUNEL) staining was performed. Briefly, overconfluent cultures and cell aggregates were fixed in 4% PFA, embedded in paraffin, and sectioned at 5-μm thickness. Deparaffinized sections were subjected to TUNEL staining (Roche Diagnostics, Mannheim, Germany) according to the manufacturer’s instructions. DNA fragmentation of apoptotic cells was observed using a Biorevo BZ-9000 microscope (Keyence).

### Detection of apoptotic cells by flow cytometry with Annexin V/PI staining

XFM and SCM cells (5 × 10^3^ cells/cm^2^) were seeded into 12-multiwell plates and cultured for 14 days. The cultured cells were collected using trypsin–EDTA on day 6, when they were considered to be in the logarithmic growth phase, and on day 14 in the overconfluent phase. Apoptotic cells were determined by flow cytometry using the Annexin V/PI system, as already described. The results were expressed as the ratio of the number of the apoptotic cells on day 14 to that on day 6.

### Bromodeoxyuridine staining

Cell-proliferative potential was monitored by bromodeoxyuridine (BrdU) staining. Both types of cells were seeded at 5 × 10^3^ cells/cm^2^ in four-well chamber slides (Thermo Fisher Scientific). On days 6 and 14 post seeding, 10 μM BrdU (Merck KGaA) was added to the cultures, which were then incubated for 3 h at 37 °C prior to fixation. BrdU incorporation was determined with mouse monoclonal anti-BrdU primary antibody (diluted 1:200; Roche Diagnostics) and Alexa 488-conjugated goat polyclonal anti-mouse IgG secondary antibody (diluted 1:1000; Life Technologies, Carlsbad, CA, USA). BrdU-positive cells were counted in 10 random fields under a confocal laser-scanning microscope (LSM 700; Carl Zeiss).

### Senescence-associated β-galactosidase staining

To examine senescence-associated β-galactosidase (SA β-gal activity), cultured cells were stained using a senescence cell histochemical staining kit (Merck KGaA) according to the manufacturer instructions. Briefly, overconfluent cultures on day 14 were subcultured in 60-mm culture dishes. The cell aggregates after SA β-gal staining were examined using a Biorevo BZ-9000 microscope (Keyence).

### Periodic acid–Schiff staining

To identify lipofuscin granules, cultured cells were stained by Periodic acid–Schiff (PAS) staining, as reported previously [30]. Briefly, cell aggregates derived from subcultured overconfluent cells were fixed in 4% PFA, embedded in paraffin, and sectioned at 5-μm thickness. Deparaffinized sections were subjected to PAS staining, and images were obtained using an Axio imager M2 microscope (Carl Zeiss).

### Statistical analysis

Statistical analyses were performed using IBM SPSS statistics software (version 23.0; IBM Japan, Tokyo, Japan). All experiments were repeated independently in triplicate, and values were expressed as the mean ± standard deviation (SD). For two-group comparisons, an unpaired *t* test or a Mann–Whitney *U* test for nonparametric data was used. The chi-square test was used to compare the presence of BrdU-positive cells. For all statistical analyses, *P* < 0.01 indicated a statistically significant difference.

## Results

### Primary culture and morphological observation of XFM cells

**Fig. 1:**
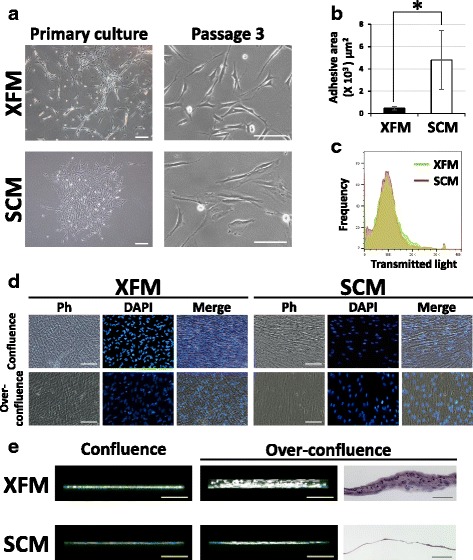
Morphological appearance and morphometric analysis of DPSCs under xenogeneic serum-free or FBS-containing culture conditions. **a** Phase-contrast images of DPSCs cultured in XFM or SCM in primary culture. Scale bars, 200 μm. Passage 3: scale bars, 100 μm. **b** Cell morphometric evaluation of adhesive areas of XFM and SCM cells at passage 3. *P < 0.01. **c** Flow-cytometric analysis of single-cell size in XFM and SCM cellular suspensions. **d** Phase-contrast (Ph) and fluorescence-microscope images of DAPI-labeled XFM and SCM cells at confluence on day 10 or overconfluence on day 14 post seeding. Scale bars, 100 μm. **e** 3D fluorescence imaging and HE staining of confluent and overconfluent cultures of Phalloidin-labeled XFM and SCM cells. Scale bars, 100 μm. SCM xenogeneic serum-containing culture medium, XFM xenogeneic serum-free culture medium, DAPI 4′,6-diamidino-2-phenylindole

Dental pulp cells isolated enzymatically from the dental pulp tissue of extracted teeth that had been transported in FBS-free basic culture medium were cultured in FBS-free culture medium (XFM) as a primary culture. Notably, the duration required to show cell division of adhered cells in primary cultured XFM cells was significantly longer than that required to form a so-called cell colony from primary culture of SCM cells on fibronectin-precoated culture dishes (9.0 ± 1.8 vs 6.0 ± 1.7 days; *n* = 6; *P* = 0.012) and on noncoated culture dishes (12.0 ± 1.9 vs 6.0 ± 1.9 days; *n* = 6; *P* = 0.001). After showing stable cell growth in primary culture, XFM cells showed active proliferation and appeared to form a multitude of small foci of accumulated cells surrounded by many solitary cells, whereas SCM cells formed cell colonies that were likely derived from single cells (Fig. 1a). Following conventional subculture, subconfluent XFM and SCM cells exhibited a fibroblast-like morphology (Fig. 1a). Notably, XFM cells showed a thin, spindle-shaped morphology with longer cell processes, regardless of the use of nonfibronectin-precoated dishes (Additional file 1: Figure S1) as compared with SCM cells. Quantitatively, the adhesive area of XFM cells was significantly smaller than that of SCM cells (*P* < 0.01; Fig. 1b). However, flow-cytometric analysis indicated similar single-cell sizes in XFM and SCM cell suspensions (Fig. 1c).

Upon reaching confluence on day 10 post seeding, XFM and SCM cells showed similar confluent monolayers according to phase-contrast and fluorescence-microscope observation (Fig. 1d). Intriguingly, when both cultures reached overconfluence on day 14, XFM cells exhibited a thicker, multilayered appearance than did SCM cells (Fig. 1d). To visualize the fine structure of overconfluent cell cultures, 3D fluorescence-image analysis was conducted using a confocal laser-scanning microscope. 3D imaging revealed that Phalloidin-labeled XFM cells formed extensive, thick multilayers, whereas SCM cells maintained a monolayer structure (Fig. 1e and Additional file 2: Movie S1). HE staining confirmed the multilayered and monolayered structures formed by XFM and SCM cells, respectively (Fig. 1e). Notably, overconfluent SCM cells cultured on fibronectin-precoated culture dishes formed a monolayered structure (Additional file 3: Figure S2). To confirm whether XFM cells with multilayered appearances on day 14 caused neoplastic transformation, ECLIA was performed using the conditioned media collected from both types of cells. As a result, we detected no common neoplastic/tumorigenic markers (CEA, SCC, or NSE) in either cell culture.


Additional file 2: Movie S1.showing 3D fluorescence image of overconfluent multilayered XFM cells (left) and monolayered SCM cells (right) (MP4 19231 kb)


### In-vitro and in-vivo stem cell characterization of XFM cells

**Fig. 2:**
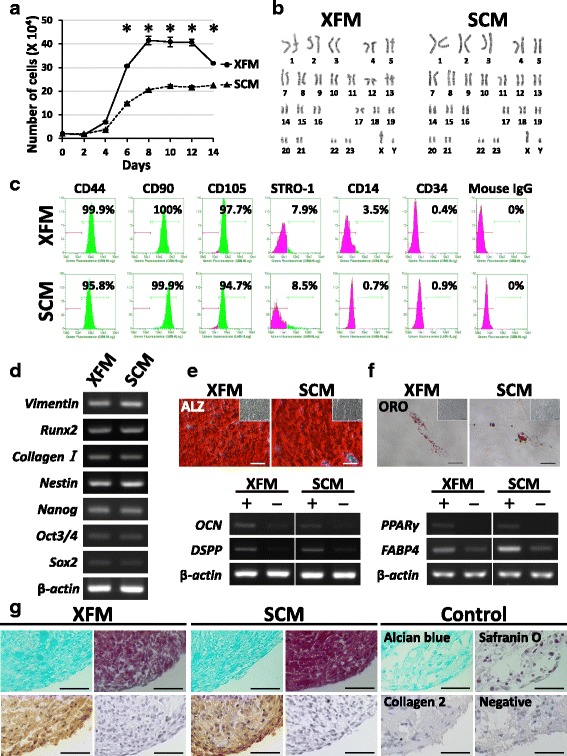
Stem cell characterization of DPSCs under xenogeneic serum-free or FBS-containing culture conditions. **a** Growth-curve evaluation of DPSCs cultured in XFM or SCM at passage 3 during 14 days of culture. *P < 0.01. **b** Karyotype analysis of XFM and SCM cells at passage 10. **c** Flow cytometry for cell-surface markers of MSCs and hematopoietic cells on XFM and SCM cells. **d** Gene-expression profile of MSC and osteo/odontogenic markers in XFM and SCM cells determined by RT-PCR. **e** Alizarin Red staining (ALZ) and RT-PCR results for osteo/odontogenic marker genes from mineral-inducing cultures of XFM and SCM cells after a 4-week induction (+) or 4 weeks without induction (−). Insets in ALZ images show no-induction cultures (4 weeks). Scale bars, 100 μm. **f** Oil Red O-staining (ORO) showing lipid droplets and RT-PCR results for adipogenic marker genes (−) in XFM and SCM cells after a 4-week adipogenic induction (+) or 4 weeks without induction (−). Insets in ORO images show no-induction cultures (4 weeks). Scale bars, 50 μm. **g** Alcian Blue, Safranin O, and immunohistochemical staining showing chondrogenic induction cultures of XFM and SCM cells after 4 weeks. No chondrogenic induction was observed after 4 weeks (control). Scale bars, 50 μm. SCM xenogeneic serum-containing culture medium, XFM xenogeneic serum-free culture medium, Runx2 runt-related transcription factor 2, Oct3/4 POU class 5 homeobox 1 (POU5F1), SOX2 sex determining region Y-box 2, OCN osteocalcin, DSPP dentin sialophosphoprotein, PPARγ, peroxisome proliferator-activated receptor gamma, FABP4 fatty acid binding protein 4

We previously established a unique in-vitro cytotoxic susceptibility test of cultured cells under serum-free culture conditions, in addition to characterizing common MSC properties [28]. First, we demonstrated in-vitro stem cell properties of XFM and SCM cells, including active cell growth (Fig. 2a), a normal karyotype (Fig. 2b), global gene/protein expression associated with the MSC phenotype (Fig. 2c, d), and trilineage differentiation potential, such as osteogenic, adipogenic, and chondrogenic lineages (Fig. 2e–g). Notably, the PDT of XFM cells was 20.1 ± 2.7 h (*n* = 3), which was markedly shorter than that of SCM cells (28.5 ± 3.7 h; *n* = 3). Collectively, these data indicated that XFM cells possessed high proliferative ability and typical MSC characteristics along with chromosomal stability.

**Fig. 3:**
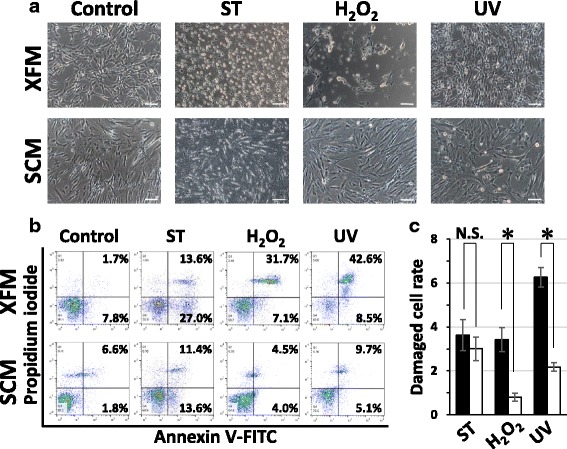
In-vitro assessment of cellular stress/damage of DPSCs induced by extrinsic cytotoxic stimuli under xenogeneic serum-free or FBS-containing culture conditions. **a** Morphological changes of DPSCs cultured in XFM and SCM before (control) and after treatment with staurosporine (ST), H_2_O_2_, or UV radiation. Scale bars, 100 μm. **b** Flow-cytometric analysis of cytotoxic stimulus-treated XFM and SCM cells using an Annexin V/PI system. **c** Quantification of the damaged cells cultured in XFM (black columns) and SCM (white columns). *P < 0.01. N.S. no significant difference, H_2_O_2_ hydrogen peroxide, UV ultraviolet, SCM xenogeneic serum-containing culture medium, XFM xenogeneic serum-free culture medium

Second, we assessed the cellular susceptibility of XFM and SCM cells to cytotoxic stimuli. As a positive control for cell damage, staurosporine affected both types of cells to the same degree, as expected. After H_2_O_2_ and UV radiation, both cell types showed degenerative changes in cell morphology (Fig. 3a). Flow cytometry with Annexin V/PI staining quantitatively indicated that the damaged cells cultured in XFM were significantly higher in number than those cultured in SCM after H_2_O_2_ and UV treatments (*P* < 0.01; Fig. 3b, c).

**Fig. 4:**
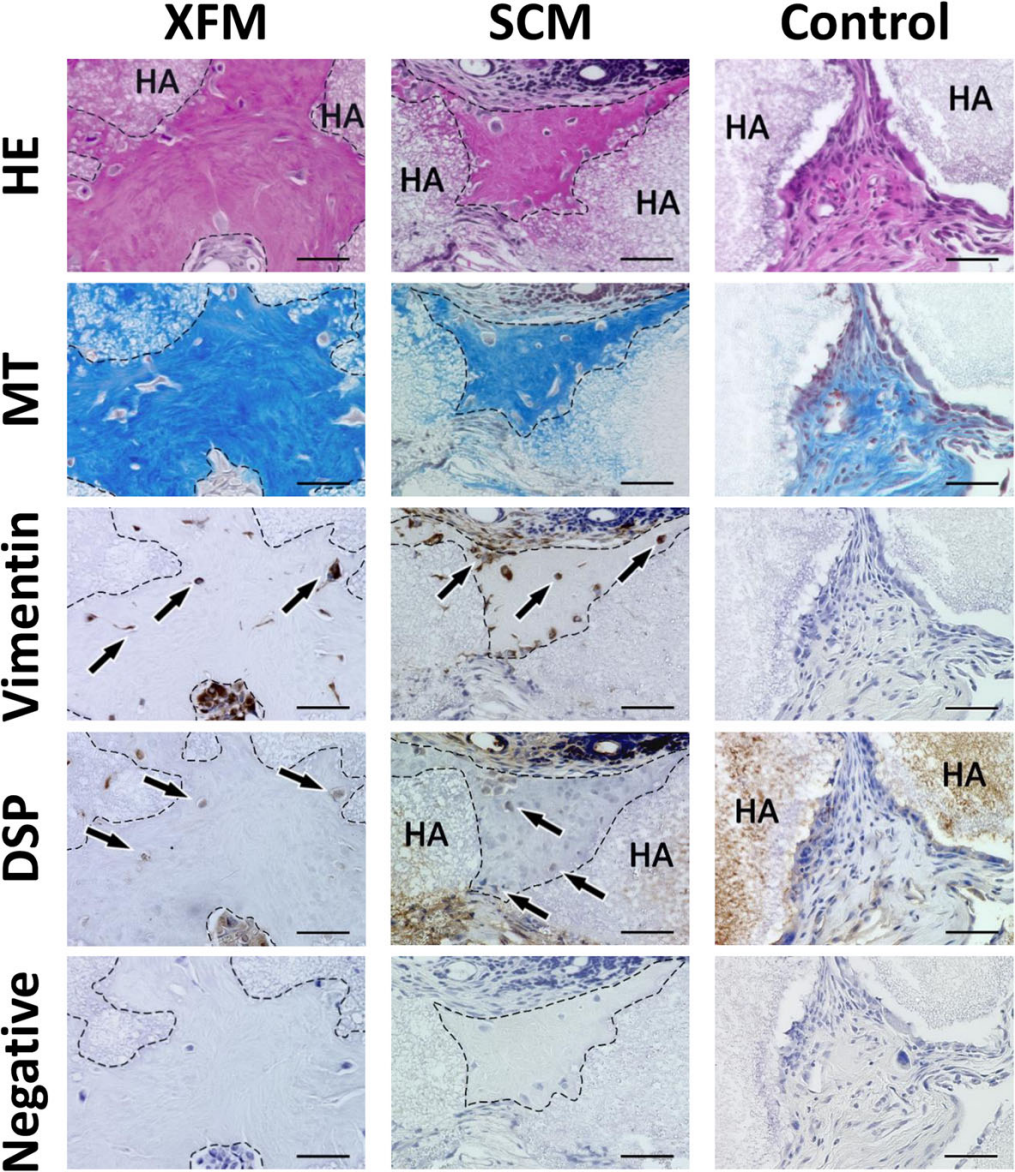
In-vivo subcutaneous transplantation of ex-vivo-expanded DPSCs cultured in xenogeneic serum-free or FBS-containing culture medium. HA scaffolds containing DPSCs cultured in XFM, SCM, or HA alone (control) were evaluated histologically by HE, MT, and immunohistochemical staining 16 weeks after transplantation. Arrows indicate cells embedded within the newly formed hard tissue, which is outlined by dashed lines. Primary antibody was omitted during immunostaining (negative control). Scale bars, 50 μm. DSP dentin sialoprotein, SCM xenogeneic serum-containing culture medium, XFM xenogeneic serum-free culture medium, HE hematoxylin and eosin, MT Masson’s trichrome, HA hydroxyapatite/β-tricalciumphosphate

Finally, we performed an in-vivo transplantation experiment using ex-vivo-expanded DPSCs cultured in XFM and SCM. HE and MT staining indicated that both types of cell/HA constructs that were transplanted subcutaneously showed osteo-dentin hard-tissue formation 16 weeks after transplantation, whereas the HA constructs alone (without cells) did not form such hard tissue (Fig. 4). Tumor formation was not observed in any of the samples. Immunohistochemistry demonstrated that the cells within newly formed hard tissue stained intensely with anti-vimentin and anti-DSP antibodies in both cell/HA constructs, whereas no positive immunoreactivity was observed in the HA constructs (Fig. 4). The immunoreactivity of the vimentin antibody was mainly observed in mesenchymal stromal cells within native dental pulp and alveolar bone, whereas DSP was preferentially observed in odontoblasts in the dental pulp, but not in osteocytes within the alveolar bone (Additional file 4: Figure S3).

### Overconfluent XFM cells show disrupted proliferative behavior

**Fig. 5:**
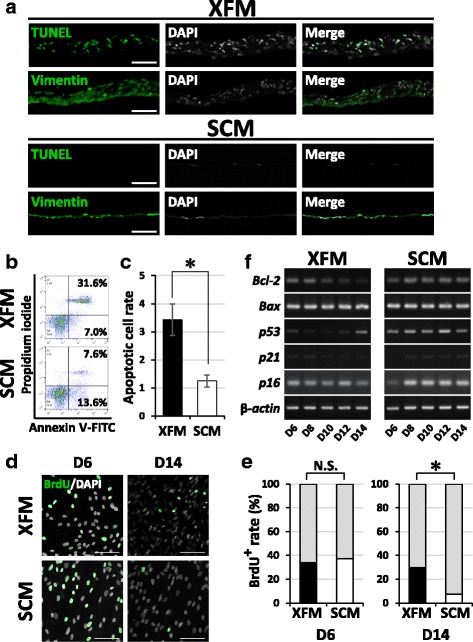
Cellular behavior of DPSCs at overconfluence under xenogeneic serum-free or FBS-containing culture medium. **a** TUNEL and immunohistochemical staining of overconfluent DPSC cultures in XFM and SCM. Scale bars, 100 μm. **b** Flow-cytometric analysis of overconfluent XFM and SCM cultures using an Annexin V/PI system. **c** Quantification of the apoptotic cells. *P < 0.01. **d** BrdU staining. **e** Quantification of BrdU-positive cells in XFM and SCM cultures on day 6 (D6) and day 14 (D14) post seeding. Scale bars, 100 μm. *P < 0.01. **f** Time-course gene-expression profile for apoptosis and cell cycle regulators during cell-growth evaluation of XFM and SCM cells as determined by RT-PCR. SCM xenogeneic serum-containing culture medium, XFM xenogeneic serum-free culture medium, DAPI 4′,6-diamidino-2-phenylindole, TUNEL terminal deoxynucleotidyl transferase-mediated deoxyuridine triphosphate nick-end labeling, Bcl-2 B-cell lymphoma 2, BAX B-cell lymphoma-2 associated X, BrdU bromodeoxyuridine, N.S. no significant difference

From the growth curves presented in Fig. 2a, we found that the number of XFM cells declined as of day 14 when the cultures reached the overconfluent state (Fig. 1e). By contrast, SCM cell growth showed a sustained plateau at this time point (Fig. 2a). Moreover, we microscopically observed a considerable number of cells with a condensed nucleus within the multilayered XFM cells, but not within the SCM monolayers (Fig. 1d, e). To clarify the reason for the reduced number of cells and nuclear condensation in the XFM culture, we conducted a TUNEL assay using overconfluent cultures on day 14. Notably, overconfluent multilayered XFM cells contained a substantial amount of TUNEL-positive cells, whereas monolayered SCM cells were completely TUNEL-negative (Fig. 5a). Flow-cytometric analysis using Annexin V/PI staining quantitatively indicated that the apoptotic cells in XFM culture were significantly higher in number than those in SCM culture (*P* < 0.01; Fig. 5b, c).

To examine whether cell division occurred, BrdU-incorporation analysis was used. We first determined the BrdU-uptake level of cultured cells on day 6 when they reached the logarithmic growth phase. As expected, both types of cells showed nearly the same level of BrdU incorporation (N.S.; Fig. 5d, e). Surprisingly, in overconfluent XFM cells, the level of BrdU uptake on day 14 was comparable to that on day 6, whereas it was significantly decreased in SCM cells at this time point (*P* < 0.01; Fig. 5d, e). Notably, a large number of BrdU-positive cells within the multilayered XFM cells had a condensed nucleus (Additional file 5: Figure S4).

Finally, we explored time-course gene-expression profiles of apoptosis-related and cell cycle-related markers during cell growth by RT-PCR. Although both types of cells expressed the pro-apoptotic gene *Bax* at a similar level, the anti-apoptotic gene *Bcl-2* was downregulated in XFM cells as the culture progressed, whereas it maintained a steady expression level in SCM cells (Fig. 5f; Additional file 6: Figure S5). The cell cycle inhibitors *p21*, *p16*, and *p53* were expressed at lower levels in XFM as compared with those in SCM cells throughout the culture (Fig. 5f; Additional file 6: Figure S5). On day 14, *p53* was upregulated in XFM cells, which at this time point showed the multilayered structure containing extensive TUNEL-positive cells. Briefly, the reduction in the number of XFM cells on day 14 was caused by apoptotic cell death, whereas the proliferative potential in the overconfluent XFM cultures was maintained at the level seen in the logarithmic growth phase.

### Aberrant proliferative behavior of overconfluent XFM cells negatively affects subculture

**Fig. 6:**
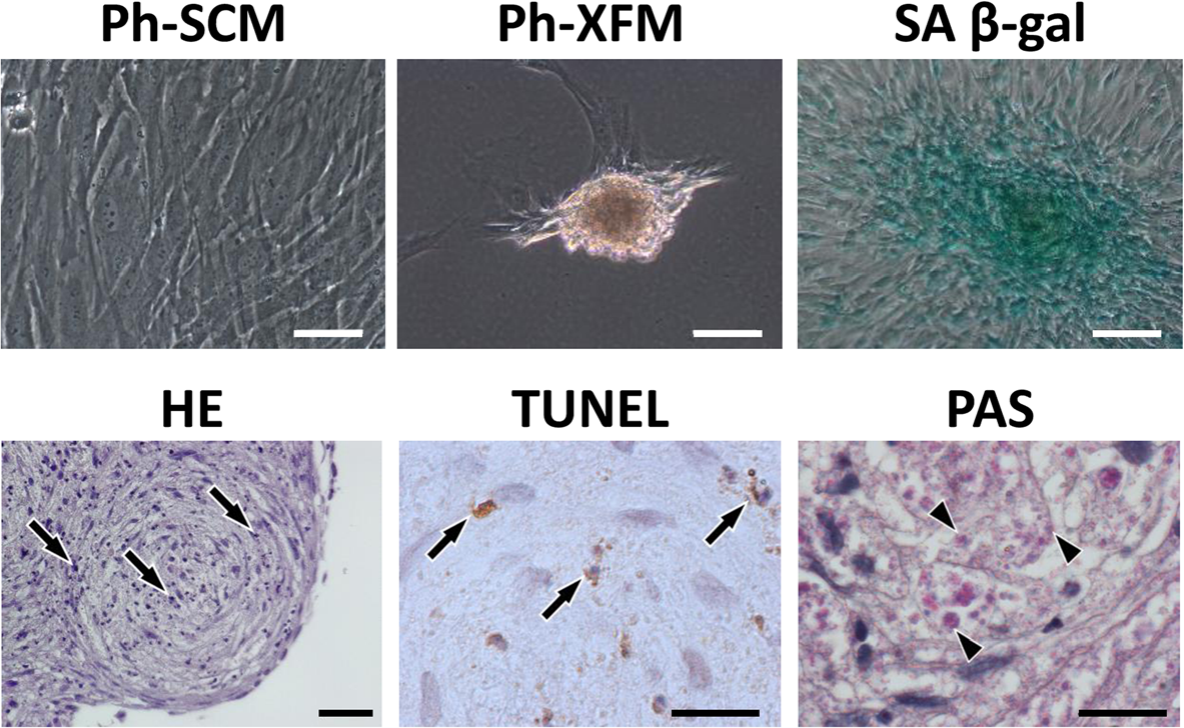
Cellular-behavioral analysis after subculture of overconfluent DPSCs cultured in xenogeneic serum-free or FBS-containing culture medium. Phase-contrast images of subcultured overconfluent monolayered SCM cells (Ph-SCM) and subcultured overconfluent multilayered XFM cells (Ph-XFM). Scale bars, 50 μm. SA β-gal staining of a cell aggregate derived from subcultured overconfluent XFM cells (SA β-gal). Scale bar, 50 μm. Histological evaluation of a cell aggregate by HE, TUNEL, and PAS staining. Arrows indicate condensed nuclei according to HE staining (scale bar, 50 μm) and TUNEL-positive cells (scale bar, 20 μm). Arrowheads indicate PAS-positive lipofuscin granules. Scale bar, 20 μm. SCM xenogeneic serum-containing culture medium, XFM xenogeneic serum-free culture medium, SA β-gal senescence-associated β-galactosidase, HE Hematoxylin and eosin, TUNEL Terminal deoxynucleotidyl transferase-mediated deoxyuridine triphosphate nick-end labeling, PAS, Periodic acid–Schiff

Next, we subcultured both types of overconfluent cultures on day 14 using a conventional trypsin–EDTA procedure. Surprisingly, XFM cells predominantly formed cell aggregates and did not proliferate further, whereas SCM cells were passaged normally and reached confluence (Fig. 6). The XFM cell aggregates were entirely positive for the cellular senescence marker SA β-gal. Moreover, HE staining indicated that the cell aggregates accumulated apoptotic cells with condensed nuclei, and the senescent cells contained perinuclear lipofuscin granules exhibiting autofluorescence. To confirm these findings, specific histochemical staining for apoptosis or lipofuscin granules was performed. TUNEL-positive/condensed nuclei and PAS-positive small granules were clearly observed within the XFM cell aggregates (Fig. 6).

### Cryopreservation does not affect the stem cell characteristics and cellular behaviors of XFM cells

We obtained comparable results for all of the aforementioned experiments using cryopreserved XFM cells (Additional files 7, 8, 9, 10, 11 and 12: Figures S6–S11).

## Discussion

Reliable ex-vivo isolation and expansion of donor-derived MSCs are required for establishing practical cell therapies. The supplementation of culture media with xenogeneic serum, including FBS, is widely accepted and believed to be essential for facilitating primary cell adhesion and subsequent cell growth in MSC culture. Although several FBS-free culture media have been developed and are widely used for the expansion of subcultured MSCs after passaging [31–33], no studies have investigated whether the FBS-free culture condition is suitable for isolation and primary culture of MSCs derived from dental pulp tissue, except for one study that reported its usability for primary isolation of a neurogenic cell population from dental pulp [34].

In this study, we examined whether the MSC population can be isolated from dental pulp tissue using cell isolation involving FBS-free basic medium for transporting extracted teeth, and whether a commercially available FBS-free culture medium is suitable for primary culture and further expansion of DPSCs. The results showed that the significantly thinner and spindle-shaped XFM cells could be isolated in primary culture and then routinely subcultured. Interestingly, although primary cultured XFM cells took a significantly longer time to adhere to the culture dishes than did SCM cells, subcultured XFM cells showed significantly greater proliferation than did SCM cells. Moreover, ex-vivo-expanded XFM cells exhibited typical MSC characteristics, including marker gene/protein expression, trilineage differentiation potential, and hard, osteo-dentin tissue formation. Additionally, long-term-cultured XFM cells maintained a normal karyotype in vitro and nontumorigenic potential in vivo. These results suggested that DPSCs can be reliably and safely isolated and expanded in vitro under FBS-free culture conditions.

However, this study revealed multiple safety concerns when handling DPSCs under FBS-free culture conditions. First, XFM cells were more susceptible to damage, as determined using the extrinsic cytotoxic stimuli H_2_O_2_ and UV light, suggesting that culture medium without FBS does not protect cultured DPSCs against extrinsic cytotoxicity, which is consistent with the cytotoxic susceptibility of periodontal ligament-derived MSCs reported previously [28]. Second, excessive cultivation (i.e., overconfluency of XFM cells) should be avoided, as this resulted in a reduction of the number of XFM cells that can be obtained at one time and the disruption of the proliferative behavior of XFM cells that form a multilayered structure consisting of both considerable TUNEL-positive apoptotic cells and BrdU-positive proliferating cells. Intriguingly, this aberrant proliferative behavior affected subcultured XFM cells after passaging at the overconfluent state. Thirdly, subcultured overconfluent XFM cells predominantly formed cell aggregates consisting of replicatively senescent and apoptotic cells, and are indeed difficult to expand further.

The aberrant cell-proliferative behaviors appeared attributable to the substantial amounts of both TUNEL-positive and BrdU-positive cells. The apoptotic cells were confirmed by time-course gene-expression patterns as determined by RT-PCR, which revealed downregulation of anti-apoptotic *Bcl-2* and upregulation of the p53-dependent apoptotic pathway [35]. Cell cycle progression in proliferating cells depends upon the downregulation of *p21* and *p16*, which are closely related to cell cycle arrest [36]. Moreover, we frequently observed BrdU-positive cells with condensed nuclei within the multilayered XFM cultures. In general, BrdU is used to detect cells entering the S phase of the cell cycle; however, a recent study described that replicatively senescent cells are arrested in not only the G1 phase but also the G2 phase, which follows the S phase [37]. Therefore, the BrdU-positive/condensed-nucleus cells were considered to be G2-arrested senescent cells. Intriguingly, we also observed that XFM cells formed considerable accumulations in primary culture and showed active proliferation and formed overlapping layers in subcultures. By contrast, SCM cells did not form accumulations during primary cultivation or multiple layers exhibiting excessive cell death/division after reaching confluence, regardless of the use of fibronectin-precoated culture dishes, suggesting that the contribution of extracellular matrix (ECM) is not responsible for the formation of cellular accumulation and multilayers. A possible mechanism of these unique proliferative behaviors is that XFM cells lose or exhibit attenuated “contact inhibition” dependent upon cell–cell interactions, although an alternative possibility is that cellular adhesion to ECM components still remains. Therefore, although further studies are needed to clarify the possible signaling pathways involved in both the contact inhibition and cellular behaviors, the presence of xenogeneic serum components likely contributes to contact inhibition and controls cell behavior, as evident from the monolayered growth of SCM cells throughout the cell culture.

Ex-vivo-expanded DPSCs are routinely cryopreserved in cell banks until use for cell therapy in the clinic. Furthermore, a simple and reliable procedure is desired for the manipulation and therapeutic preparation of DPSCs. Hence, we independently conducted the aforementioned in-vitro and in-vivo experiments using DMSO-free cryopreserved XFM cell stocks. The results indicated that thawed XFM cells showed comparable stem cell characteristics and aberrant proliferative behaviors to those of noncryopreserved XFM cells. Therefore, cryopreservation of XFM cells would be very useful for research and clinical use. Consequently, the findings of this comprehensive study hold promise for optimal practical cell-culture protocols for handling DPSCs under xenogeneic serum-free culture conditions.

## Conclusion

This report describes the first successful isolation and expansion of an MSC population from donor-derived tissue (dental pulp) under xenogeneic serum-free culture conditions and demonstrates the suitability of serum-free culture medium for application in DMSO-free cryopreservation. Although xenogeneic serum-free cultivation facilitates effective large-scale expansion of DPSCs, excessive cultivation to overconfluence should be avoided, because this abrogates the proliferative potential and further cultivation of XFM cells. Establishing appropriate xenogeneic serum-free culture conditions in future studies will enable us to obtain clinically feasible MSCs from dental pulp tissue, which represents a readily accessible cell source for stem cell research and therapy.

## Additional files

Additional file 1: Figure S1. showing phase-contrast images of DPSCs cultured in XFM on noncoated or fibronectin-precoated culture dishes. Scale bars, 100 μm (TIF 1047 kb)

Additional file 3: Figure S2. showing HE staining of DPSCs cultured in FBS-containing medium (SCM) on fibronectin-precoated dish after reaching confluence. Scale bar, 100 μm (TIF 295 kb)

Additional file 4: Figure S3. showing histological and immunohistochemical examination of native dental pulp and alveolar bone. HE and MT staining. Scale bars, 50 μm. Asterisk denotes bone marrow. DSP dentin sialoprotein, D dentin, P dental pulp, Od odontoblasts (TIF 3813 kb)

Additional file 5: Figure S4. showing BrdU staining of overconfluent XFM cultures on day 14 post seeding. Arrow indicates BrdU-positive oval nucleus. Arrowheads indicate BrdU-positive condensed nuclei. Scale bars, 100 μm (TIF 954 kb)

Additional file 6: Figure S5. showing semiquantitative densitometric analysis of the corresponding results of RT-PCR indicated in Fig. 5f. Time-course gene-expression profile for apoptosis and cell cycle regulators during cell-growth evaluation in DPSCs cultured in XFM (black columns) and SCM (white columns). Observed signals expressed as a ratio to β-actin signal intensity for the respective genes (TIF 339 kb)

Additional file 7: Figure S6. showing stem cell characterization of cryopreserved DPSCs cultured in xenogeneic serum-free culture medium (c-XFM). **a** Growth-curve evaluation of c-XFM cells and noncryopreserved XFM cells at passage 3 during 14 days of culture. No statistically significant differences observed in cell growth during the 14 days post seeding. **b** Normal karyotype maintained in c-XFM cells at passage 10. **c** Flow cytometry for cell-surface markers of MSCs and hematopoietic cells on c-XFM cells. **d** Gene-expression profile of MSC and osteo/odontogenic markers in c-XFM cells determined by RT-PCR. **e** Alizarin Red staining (ALZ) and RT-PCR results for osteo/odontogenic marker genes from mineral-inducing cultures of c-XFM cells after a 4-week induction (+) or 4 weeks without induction (−). Insets in ALZ images show no-induction cultures (4 weeks). Scale bars, 100 μm. **f** Oil Red O-staining (ORO) showing lipid droplets and RT-PCR results for adipogenic marker genes (−) in c-XFM cells after a 4-week adipogenic induction (+) or 4 weeks without induction (−). Insets in ORO images showing no-induction cultures (4 weeks). Scale bars, 50 μm. **g** Alcian Blue, Safranin O, and immunohistochemical staining showing chondrogenic induction cultures of c-XFM cells after 4 weeks. No chondrogenic induction after 4 weeks (control). Scale bars, 50 μm (TIF 2149 kb)

Additional file 8: Figure S7. showing in-vitro assessment of cellular stress/damage of cryopreserved DPSCs induced by extrinsic cytotoxic stimuli under xenogeneic serum-free culture medium (c-XFM). **a** Degenerative morphological changes of c-XFM cells before (control) and after treatment with staurosporine (ST), H_2_O_2_, or UV radiation. Scale bars, 100 μm. **b** Flow-cytometric analysis of cytotoxic stimulus-treated c-XFM cells and DPSCs cultured in SCM using an Annexin V/PI system. **c** Quantification of the damaged cells in c-XFM (black columns) and SCM (white columns) cultures. *P < 0.01. N.S. no significant difference (TIF 1570 kb)

Additional file 9: Figure S8. showing in-vivo subcutaneous transplantation of ex-vivo-expanded cryopreserved DPSCs cultured in xenogeneic serum-free culture medium (c-XFM). HA scaffolds containing c-XFM cells were evaluated histologically by HE, MT, and immunohistochemical staining 16 weeks after transplantation. Arrows indicate cells embedded within the newly formed hard tissue, which is outlined by dashed lines. Primary antibody omitted during immunostaining (negative control). Scale bars, 50 μm. DSP dentin sialoprotein (TIF 2466 kb)

Additional file 10: Figure S9. showing cellular behavior of cryopreserved DPSCs at overconfluence under xenogeneic serum-free culture medium (c-XFM). **a** TUNEL staining was visualized positively in c-XFM cells, but not in DPSCs cultured in SCM, whereas both cell types were positively immunostained for vimentin. Scale bars, 100 μm. **b** Flow-cytometric analysis using an Annexin V/PI system indicated that the number of apoptotic cells in overconfluent c-XFM cultures was higher than those in SCM cultures. **c** Quantification of the apoptotic cells demonstrated a statistically significant difference between cell types. *P < 0.01. **d** BrdU uptake in overconfluent c-XFM cultures on day 14 was comparable to that on day 6, whereas it was decreased in SCM cells at this time point. **e** Quantification of BrdU-positive cells in c-XFM and SCM cultures statistically supported the results in **d**. Scale bars, 100 μm. *P < 0.01. **f** Time-course gene-expression profile for apoptosis and cell cycle regulators during cell-growth evaluation of c-XFM cells was comparable to that in XFM cells according to RT-PCR analysis (see Fig. 5f). N.S. no significant difference (TIF 1530 kb)

Additional file 11: Figure S10. showing semiquantitative densitometric analysis of the RT-PCR results from Additional file 10: Figure S9f. Time-course gene-expression profile for apoptosis and cell cycle regulators during cell-growth evaluation in c-XFM (black columns) and SCM (white columns) cultures. Observed signals expressed as a ratio to β-actin signal intensity of the respective genes. Expression pattern in c-XFM cells was comparable to that in XFM cells (see Fig. 5f) (TIF 337 kb)

Additional file 12: Figure S11. showing cellular-behavioral analysis after subculture of overconfluent cryopreserved DPSCs cultured in xenogeneic serum-free culture medium (c-XFM). Phase-contrast image of subcultured overconfluent, multilayered c-XFM cells (Ph-c-XFM). Scale bar, 50 μm. Positive SA β-gal staining of a cell aggregate derived from subcultured overconfluent c-XFM cells (SA β-gal). Scale bar, 50 μm. Histological evaluation of cell aggregate by HE, TUNEL, and PAS staining. Arrows indicate condensed nuclei according to HE staining (scale bar, 50 μm) and TUNEL-positive cells (scale bar, 20 μm). Arrowheads indicate PAS-positive lipofuscin granules. Scale bar, 20 μm (TIF 2270 kb)

## Abbreviations

3D: Three-dimensional
BrdU: Bromodeoxyuridine
DAPI: 4′,6-Diamidino-2-phenylindole
DMSO: Dimethyl sulfoxide
DPSC: Dental pulp stem cell
DSP: Dentin sialoprotein
EDTA: Ethylenediaminetetraacetic acid
FBS: Fetal bovine serum
H_2_O_2_: Hydrogen peroxide
HA: Hydroxyapatite/β-tricalciumphosphate
HE: Hematoxylin and eosin
Ig: Immunoglobulin
MSC: Mesenchymal stem cell
MT: Masson’s trichrome
PAS: Periodic acid–Schiff
PBS: Calcium and magnesium-free phosphate-buffered saline
PDT: Population doubling time
PFA: Paraformaldehyde
PI: Propidium iodide
RT-PCR: Reverse-transcription polymerase chain reaction
SA β-gal: Senescence-associated β-galactosidase
SCM: Xenogeneic serum-containing culture medium
SD: Standard deviation
TUNEL: Terminal deoxynucleotidyl transferase-mediated deoxyuridine triphosphate nick-end labeling
UV: Ultraviolet
XFM: Xenogeneic serum-free culture medium
