# Supplementary figures and images for: Establishment of xenogeneic serum-free culture methods for handling human dental pulp stem cells using clinically oriented in-vitro and in-vivo conditions
Source: Stem Cell Res Ther. 2018 Feb 3;9:25. doi: 10.1186/s13287-017-0761-5 (PMC5797401; doi:10.1186/s13287-017-0761-5)

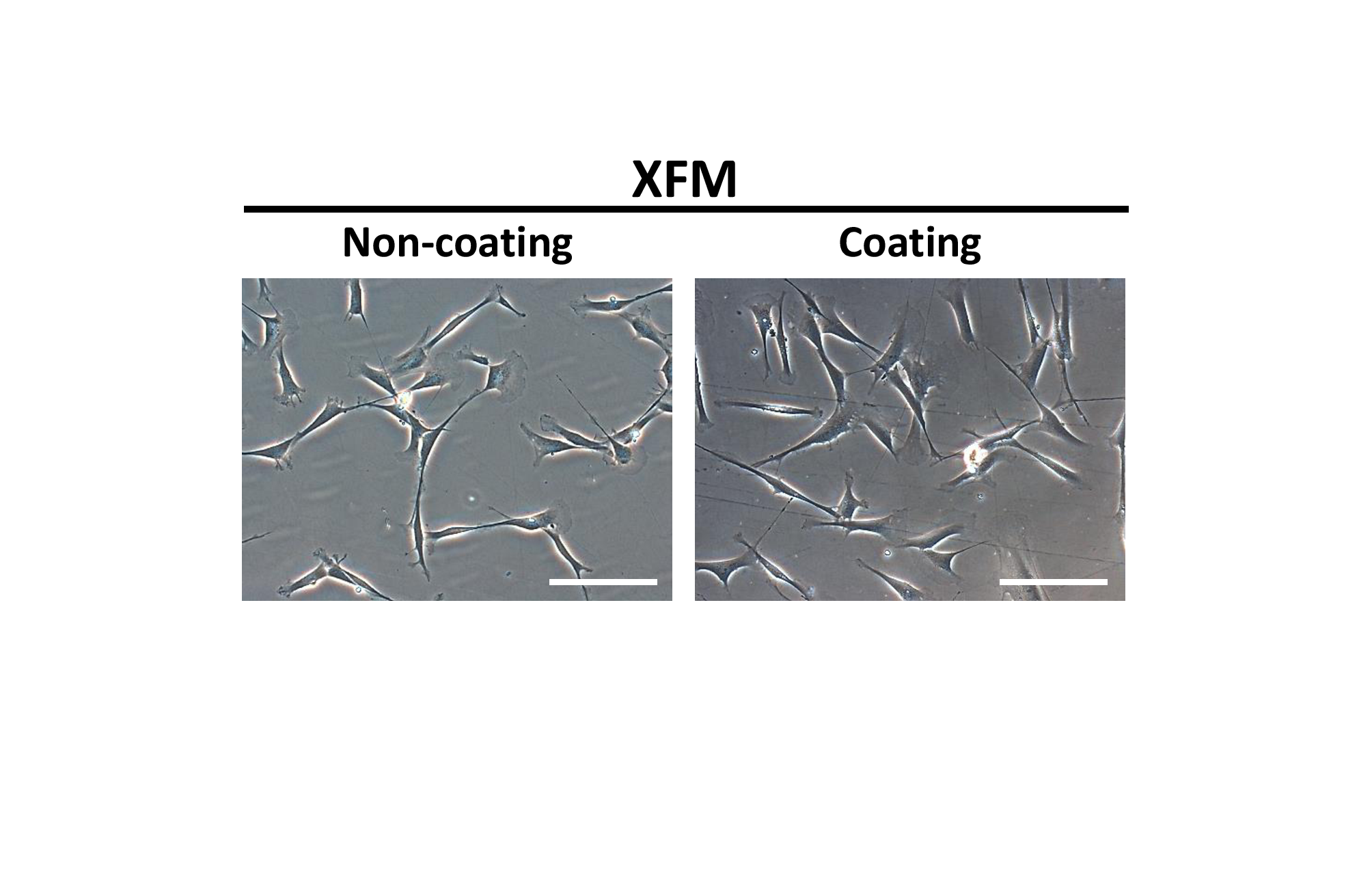

Supplement: Supplementary file 2 — showing phase-contrast images of DPSCs cultured in XFM on noncoated or fibronectin-precoated culture dishes. Scale bars, 100 μm (TIF 1047 kb) [file 13287_2017_761_MOESM1_ESM.tif]

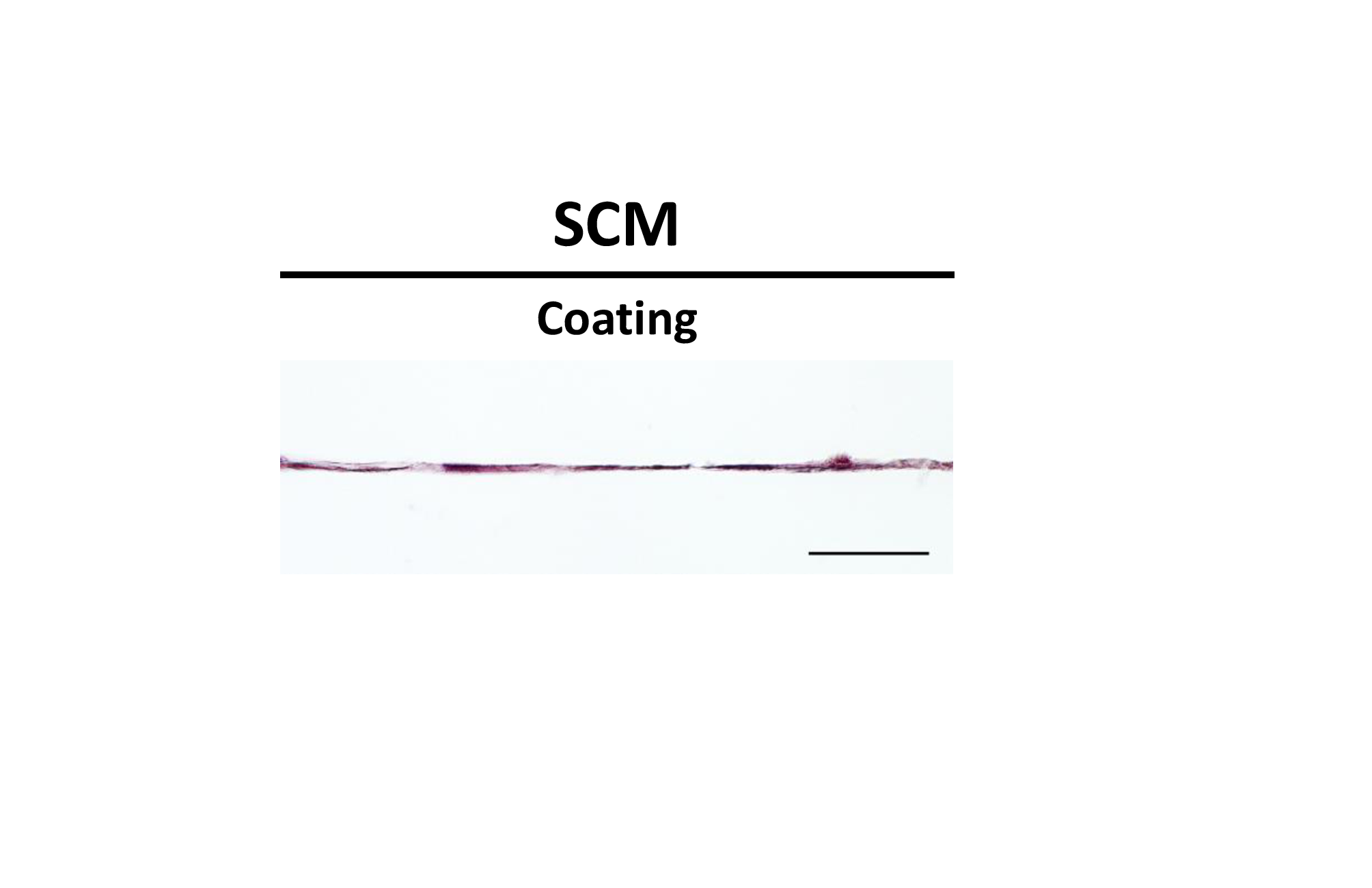

Supplement: Supplementary file 3 — showing HE staining of DPSCs cultured in FBS-containing medium (SCM) on fibronectin-precoated dish after reaching confluence. Scale bar, 100 μm (TIF 295 kb) [file 13287_2017_761_MOESM3_ESM.tif]

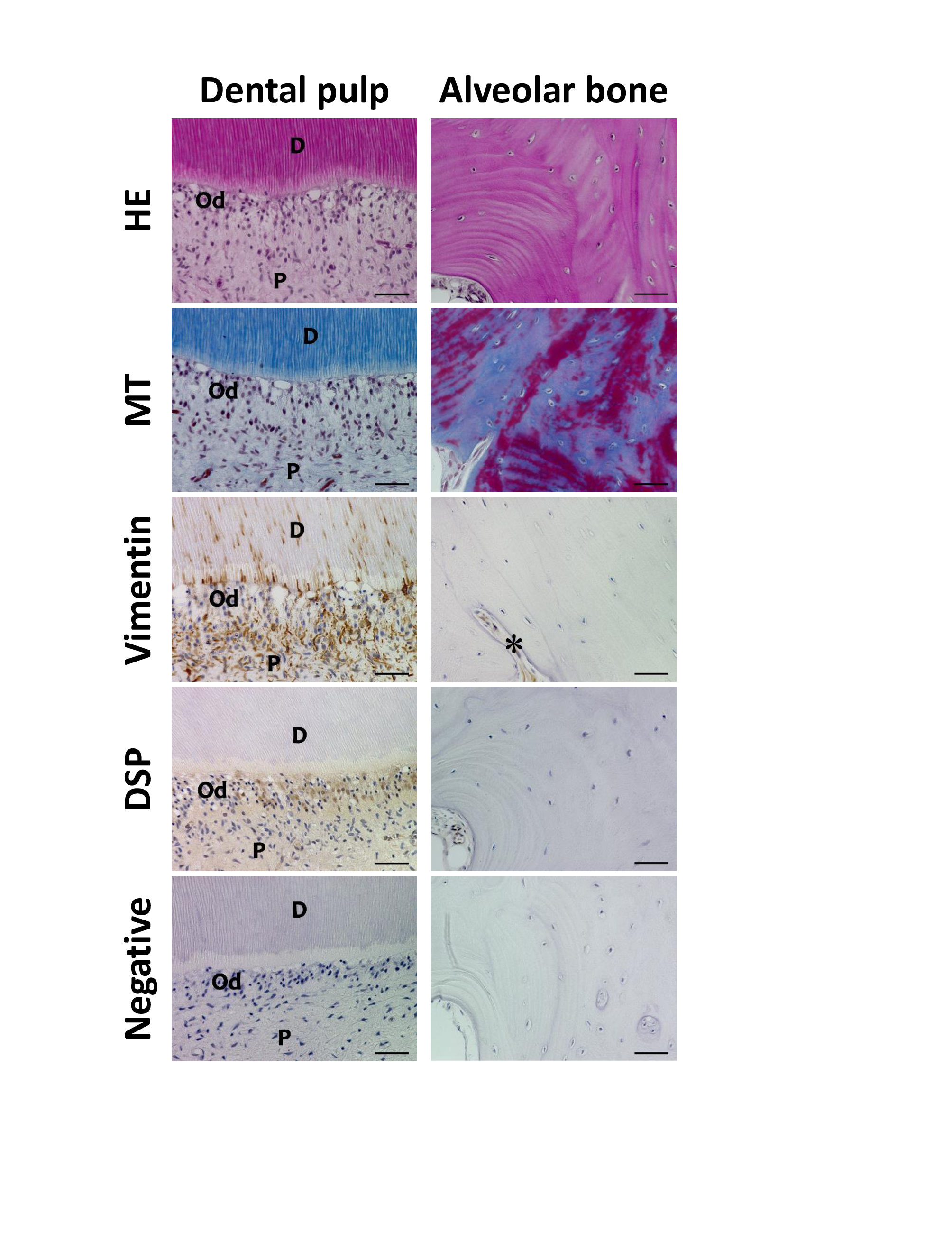

Supplement: Supplementary file 4 — showing histological and immunohistochemical examination of native dental pulp and alveolar bone. HE and MT staining. Scale bars, 50 μm. Asterisk denotes bone marrow. DSP dentin sialoprotein, D dentin, P dental pulp, Od odontoblasts (TIF 3813 kb) [file 13287_2017_761_MOESM4_ESM.tif]

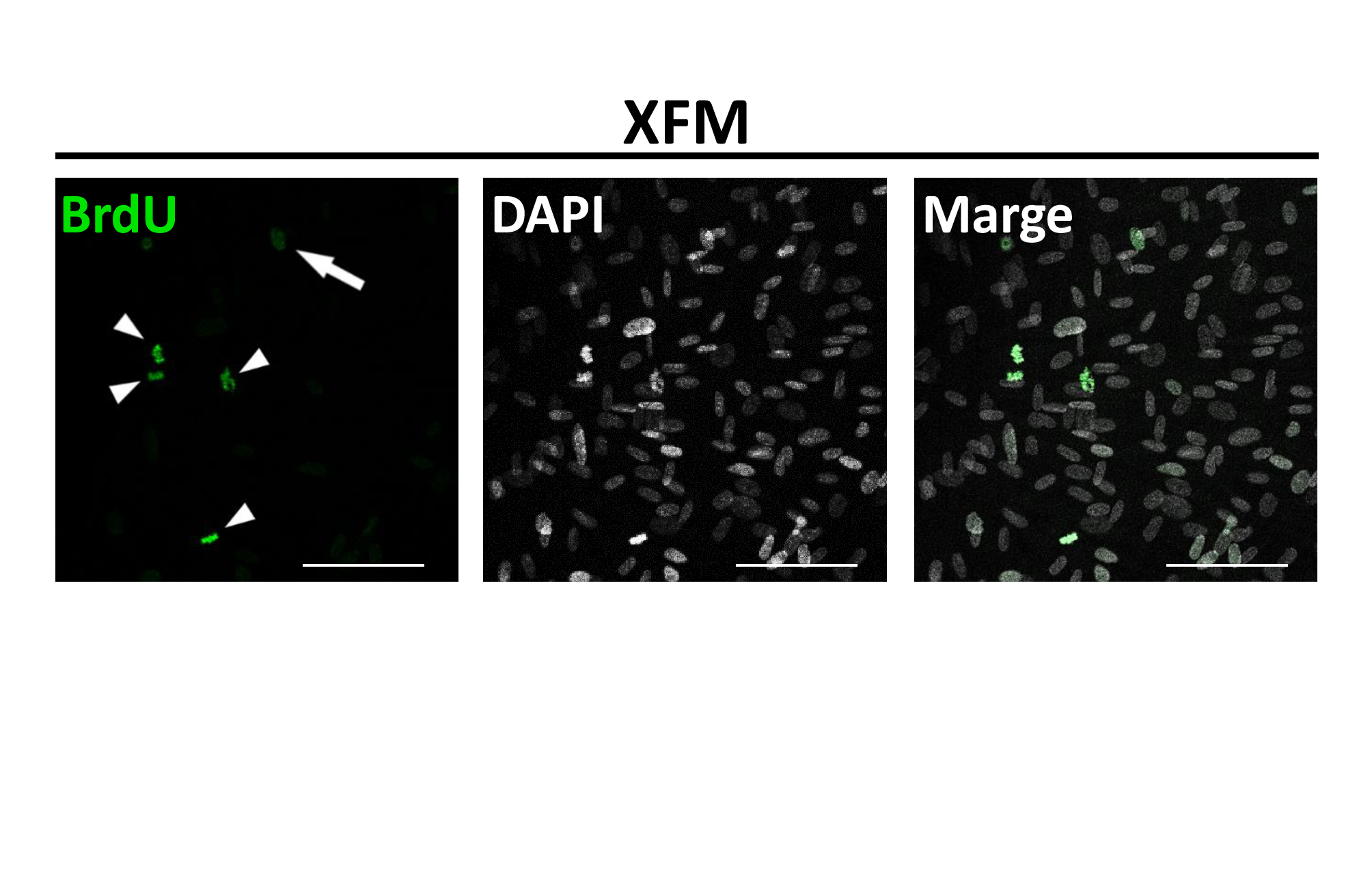

Supplement: Supplementary file 5 — showing BrdU staining of overconfluent XFM cultures on day 14 post seeding. Arrow indicates BrdU-positive oval nucleus. Arrowheads indicate BrdU-positive condensed nuclei. Scale bars, 100 μm (TIF 954 kb) [file 13287_2017_761_MOESM5_ESM.tif]

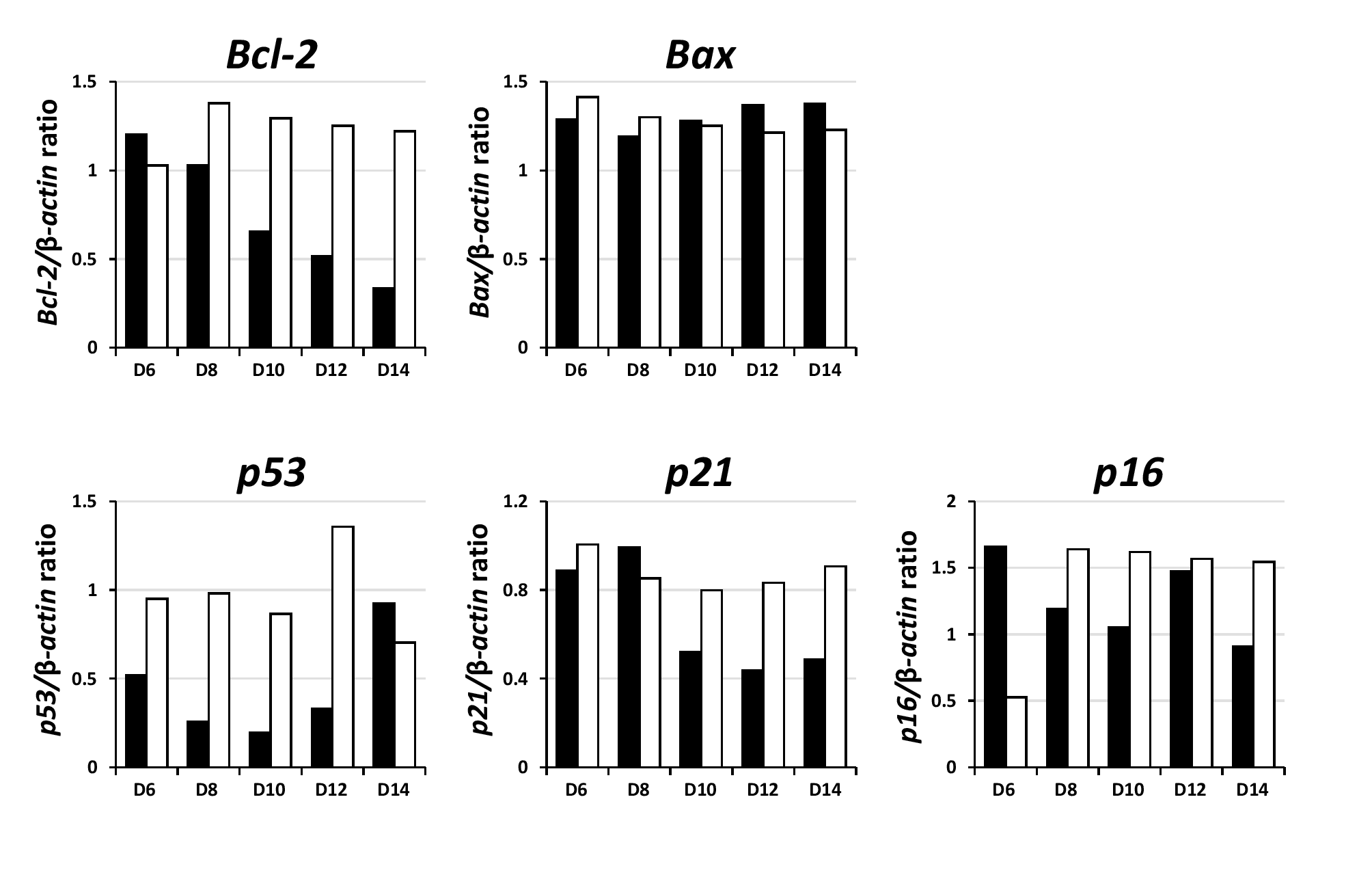

Supplement: Supplementary file 6 — showing semiquantitative densitometric analysis of the corresponding results of RT-PCR indicated in Fig. 5f. Time-course gene-expression profile for apoptosis and cell cycle regulators during cell-growth evaluation in DPSCs cultured in XFM (black columns) and SCM (white columns). Observed signals expressed as a ratio to β-actin signal intensity for the respective genes (TIF 339 kb) [file 13287_2017_761_MOESM6_ESM.tif]

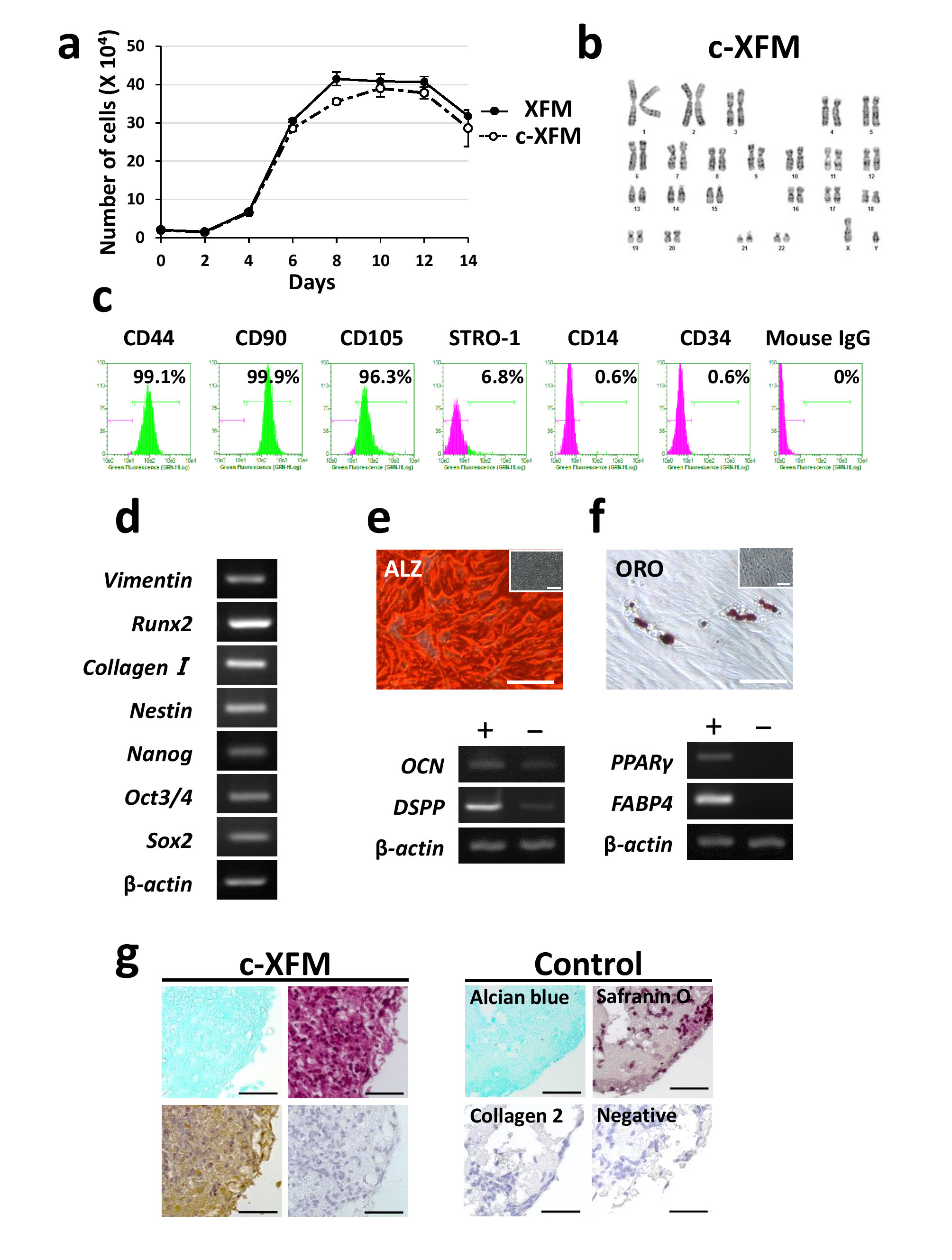

Supplement: Supplementary file 7 — showing stem cell characterization of cryopreserved DPSCs cultured in xenogeneic serum-free culture medium (c-XFM). a Growth-curve evaluation of c-XFM cells and noncryopreserved XFM cells at passage 3 during 14 days of culture. No statistically significant differences observed in cell growth during the 14 days post seeding. b Normal karyotype maintained in c-XFM cells at passage 10. c Flow cytometry for cell-surface markers of MSCs and hematopoietic cells on c-XFM cells. d Gene-expression profile of MSC and osteo/odontogenic markers in c-XFM cells determined by RT-PCR. e Alizarin Red staining (ALZ) and RT-PCR results for osteo/odontogenic marker genes from mineral-inducing cultures of c-XFM cells after a 4-week induction (+) or 4 weeks without induction (−). Insets in ALZ images show no-induction cultures (4 weeks). Scale bars, 100 μm. f Oil Red O-staining (ORO) showing lipid droplets and RT-PCR results for adipogenic marker genes (−) in c-XFM cells after a 4-week adipogenic induction (+) or 4 weeks without induction (−). Insets in ORO images showing no-induction cultures (4 weeks). Scale bars, 50 μm. g Alcian Blue, Safranin O, and immunohistochemical staining showing chondrogenic induction cultures of c-XFM cells after 4 weeks. No chondrogenic induction after 4 weeks (control). Scale bars, 50 μm (TIF 2149 kb) [file 13287_2017_761_MOESM7_ESM.tif]

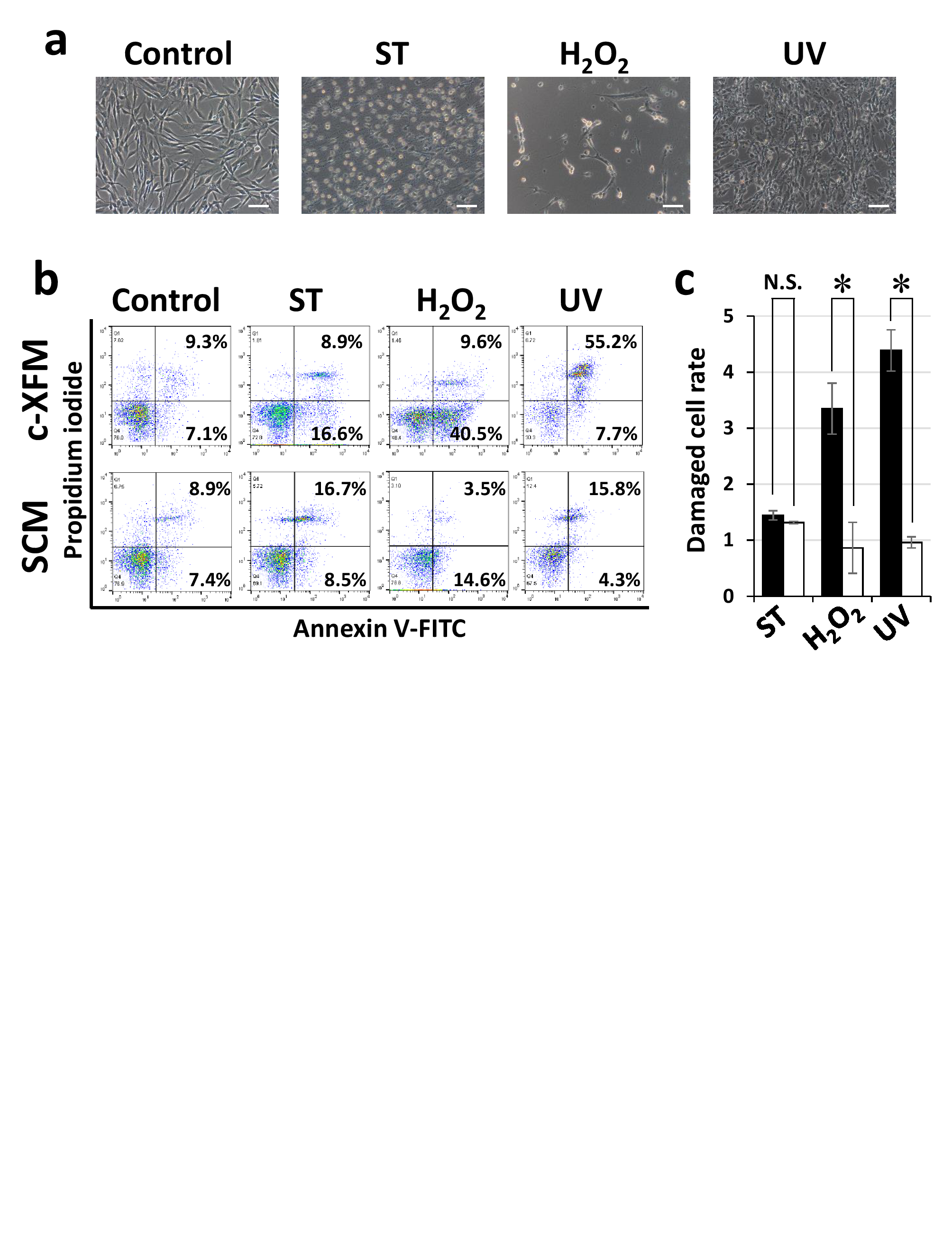

Supplement: Supplementary file 8 — showing in-vitro assessment of cellular stress/damage of cryopreserved DPSCs induced by extrinsic cytotoxic stimuli under xenogeneic serum-free culture medium (c-XFM). a Degenerative morphological changes of c-XFM cells before (control) and after treatment with staurosporine (ST), H2O2, or UV radiation. Scale bars, 100 μm. b Flow-cytometric analysis of cytotoxic stimulus-treated c-XFM cells and DPSCs cultured in SCM using an Annexin V/PI system. c Quantification of the damaged cells in c-XFM (black columns) and SCM (white columns) cultures. *P < 0.01. N.S. no significant difference (TIF 1570 kb) [file 13287_2017_761_MOESM8_ESM.tif]

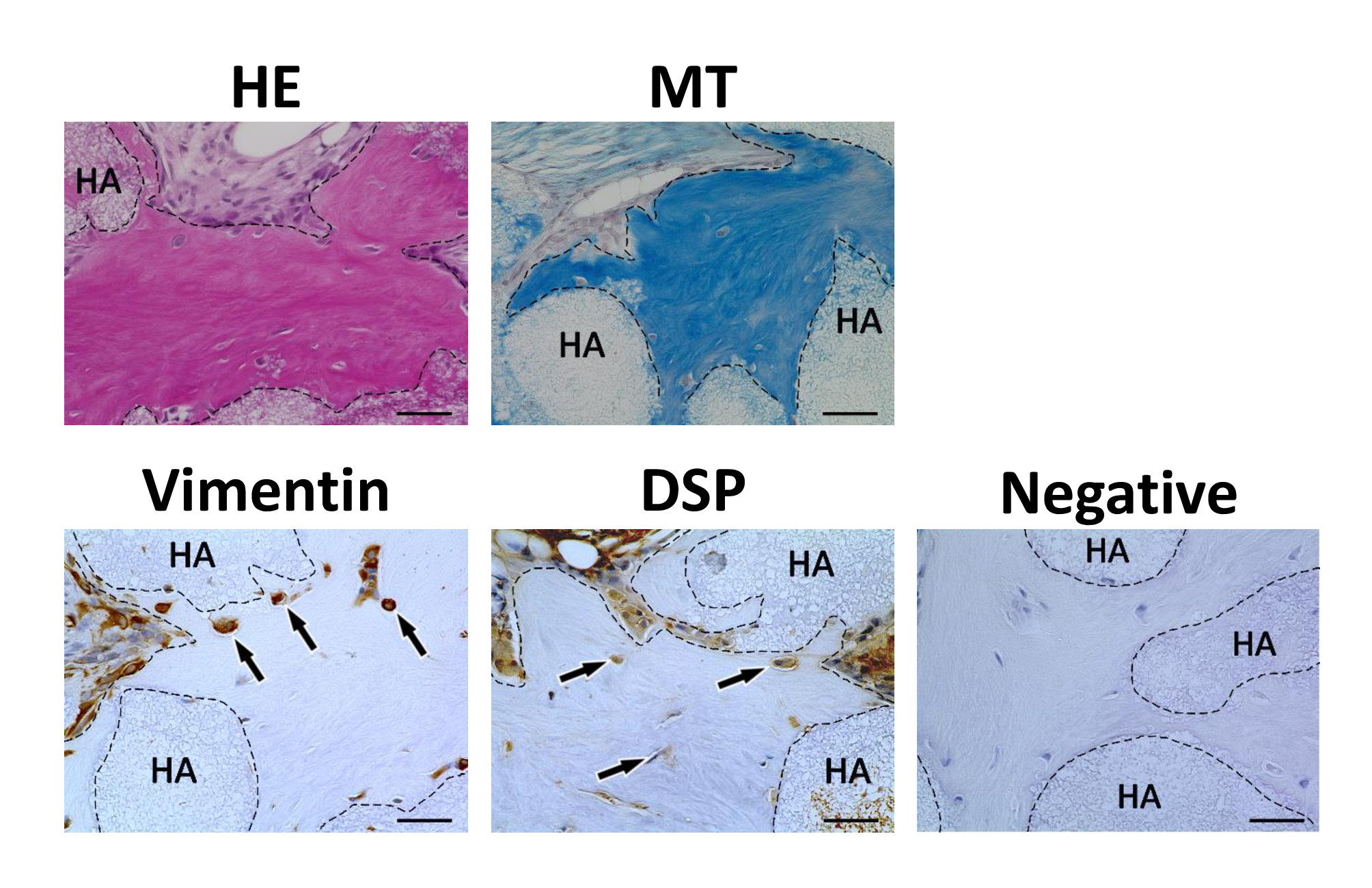

Supplement: Supplementary file 9 — showing in-vivo subcutaneous transplantation of ex-vivo-expanded cryopreserved DPSCs cultured in xenogeneic serum-free culture medium (c-XFM). HA scaffolds containing c-XFM cells were evaluated histologically by HE, MT, and immunohistochemical staining 16 weeks after transplantation. Arrows indicate cells embedded within the newly formed hard tissue, which is outlined by dashed lines. Primary antibody omitted during immunostaining (negative control). Scale bars, 50 μm. DSP dentin sialoprotein (TIF 2466 kb) [file 13287_2017_761_MOESM9_ESM.tif]

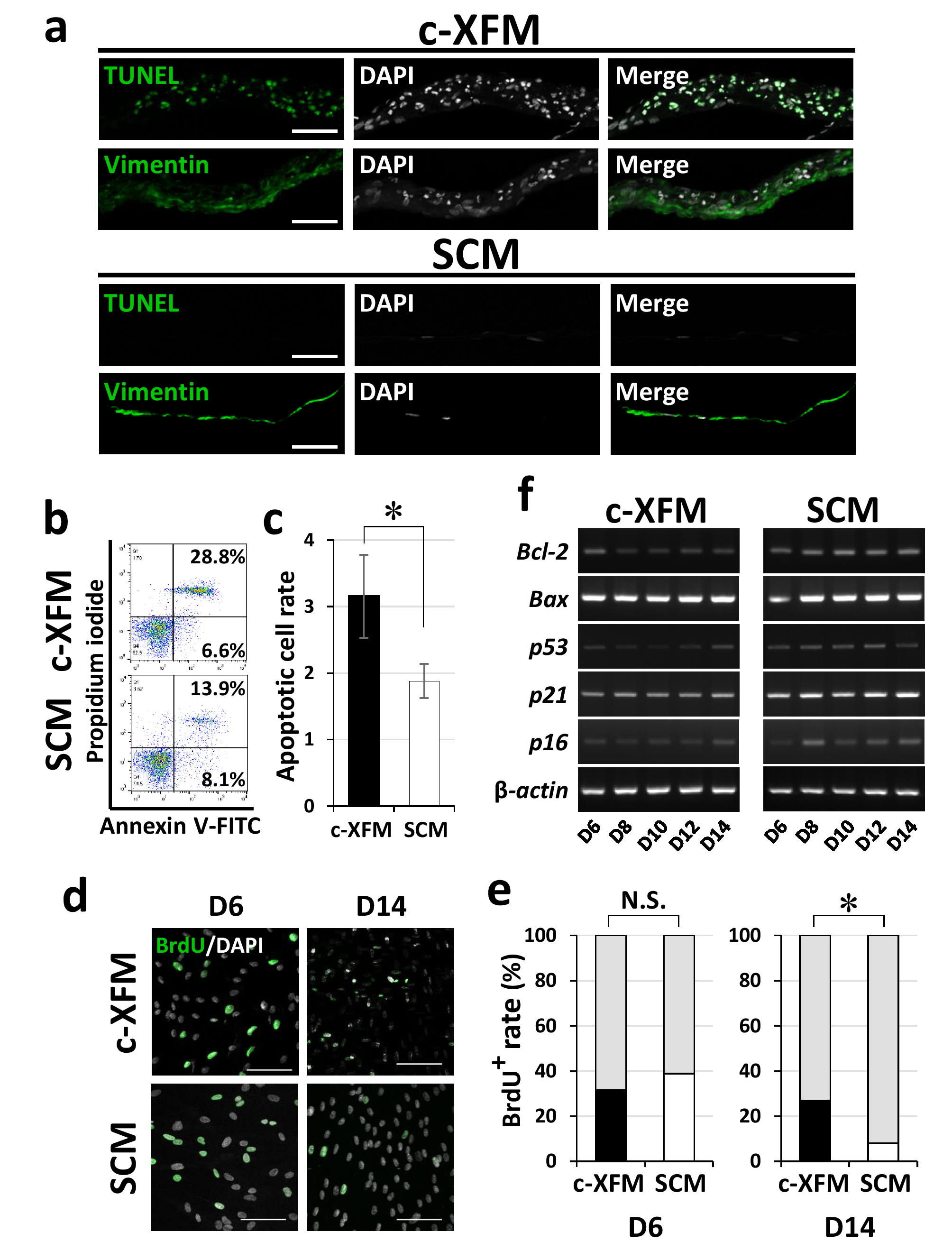

Supplement: Supplementary file 10 — showing cellular behavior of cryopreserved DPSCs at overconfluence under xenogeneic serum-free culture medium (c-XFM). a TUNEL staining was visualized positively in c-XFM cells, but not in DPSCs cultured in SCM, whereas both cell types were positively immunostained for vimentin. Scale bars, 100 μm. b Flow-cytometric analysis using an Annexin V/PI system indicated that the number of apoptotic cells in overconfluent c-XFM cultures was higher than those in SCM cultures. c Quantification of the apoptotic cells demonstrated a statistically significant difference between cell types. *P < 0.01. d BrdU uptake in overconfluent c-XFM cultures on day 14 was comparable to that on day 6, whereas it was decreased in SCM cells at this time point. e Quantification of BrdU-positive cells in c-XFM and SCM cultures statistically supported the results in d. Scale bars, 100 μm. *P < 0.01. f Time-course gene-expression profile for apoptosis and cell cycle regulators during cell-growth evaluation of c-XFM cells was comparable to that in XFM cells according to RT-PCR analysis (see Fig. 5f). N.S. no significant difference (TIF 1530 kb) [file 13287_2017_761_MOESM10_ESM.tif]

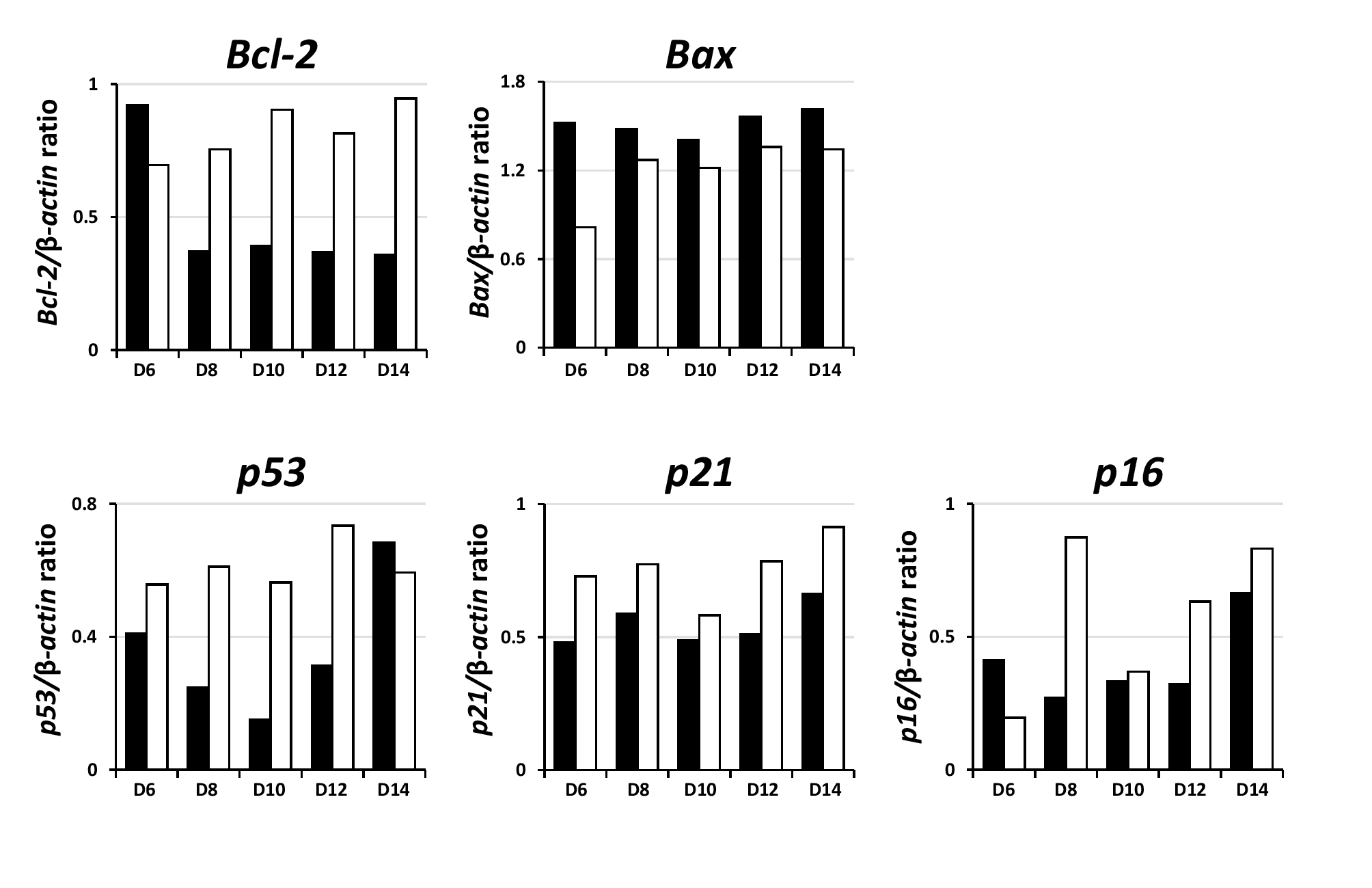

Supplement: Supplementary file 11 — showing semiquantitative densitometric analysis of the RT-PCR results from Additional file 10: Figure S9f. Time-course gene-expression profile for apoptosis and cell cycle regulators during cell-growth evaluation in c-XFM (black columns) and SCM (white columns) cultures. Observed signals expressed as a ratio to β-actin signal intensity of the respective genes. Expression pattern in c-XFM cells was comparable to that in XFM cells (see Fig. 5f) (TIF 337 kb) [file 13287_2017_761_MOESM11_ESM.tif]

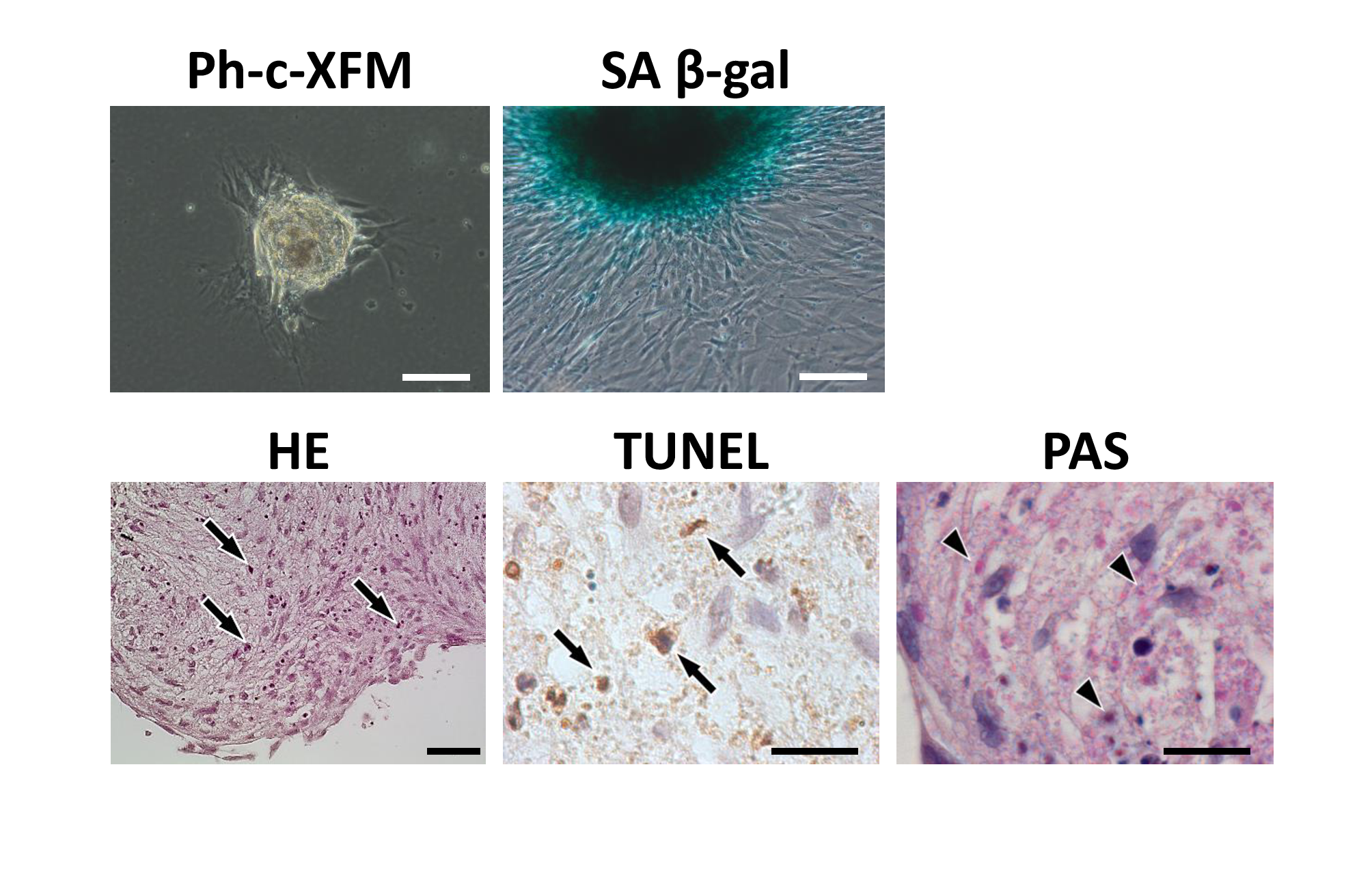

Supplement: Supplementary file 12 — showing cellular-behavioral analysis after subculture of overconfluent cryopreserved DPSCs cultured in xenogeneic serum-free culture medium (c-XFM). Phase-contrast image of subcultured overconfluent, multilayered c-XFM cells (Ph-c-XFM). Scale bar, 50 μm. Positive SA β-gal staining of a cell aggregate derived from subcultured overconfluent c-XFM cells (SA β-gal). Scale bar, 50 μm. Histological evaluation of cell aggregate by HE, TUNEL, and PAS staining. Arrows indicate condensed nuclei according to HE staining (scale bar, 50 μm) and TUNEL-positive cells (scale bar, 20 μm). Arrowheads indicate PAS-positive lipofuscin granules. Scale bar, 20 μm (TIF 2270 kb) [file 13287_2017_761_MOESM12_ESM.tif]
